# Association between integrase strand transfer inhibitor use with insulin resistance and incident diabetes mellitus in persons living with HIV: a systematic review and meta-analysis

**DOI:** 10.1136/bmjdrc-2022-003136

**Published:** 2023-02-08

**Authors:** Frank Mulindwa, Habiba Kamal, Barbara Castelnuovo, Dathan M Byonanebye, Jean-Marc Schwarz, Robert Bollinger, Nele Brusselaers

**Affiliations:** 1 Capacity Building Program, Makerere University Infectious Diseases Institute, Kampala, Uganda; 2 Global Health Institute, University of Antwerp, Antwerpen, Belgium; 3 Department of Infectious Diseases, Karolinska University Hospital, Stockholm, Sweden; 4 Department of Medicine Huddinge, Karolinska Institute, Stockholm, Sweden; 5 Biostatistics and Databases Program, Kirby Institute University of New South Wales, Sydney, New South Wales, Australia; 6 Community and Behavioral Sciences, Makerere University, Kampala, Uganda; 7 School of Medicine, University of California San Francisco, San Francisco, California, USA; 8 School of Medicine, Johns Hopkins University, Baltimore, Maryland, USA; 9 Department of Microbiology, Tumour and Cell Biology, Karolinska Institutet, Stockholm, Sweden

**Keywords:** HIV, Insulin Resistance, Metabolic Syndrome, Meta-Analysis

## Abstract

Whether integrase strand transfer inhibitors (INSTIs) are associated with a higher risk of incident type 2 diabetes mellitus (DM) than other antiretroviral therapies (ART) needs to be established.

MEDLINE, Embase, Web of Science, and ClinicalTrials.gov registries were searched for studies published between 1 January 2000 and 15 June 2022. Eligible studies reported incident DM or mean changes in insulin resistance measured by Homeostatic Model for Insulin Resistance (HOMA-IR) in patients on INSTIs compared with other ARTs. We performed random-effects meta-analyses to obtain pooled relative risks (RRs) with 95% CIs.

A total of 16 studies were pooled: 13 studies meta-analyzed for incident diabetes with a patient population of 72 404 and 3 for changes in HOMA-IR. INSTI therapy was associated with a lower risk of incident diabetes in 13 studies (RR 0.80, 95% CI 0.67 to 0.96, I^2^=29%), of which 8 randomized controlled trials demonstrated a 22% reduced risk (RR 0.88, 95% CI 0.81 to 0.96, I^2^=0%). INSTIs had a lower risk compared with non-nucleoside reverse transcriptase inhibitors (RR 0.75, 95% CI 0.63 to 0.89, I^2^=0%) but similar to protease inhibitor-based therapy (RR 0.78, 95% CI 0.61 to 1.01, I^2^=27%). The risk was lower in studies with longer follow-up (RR 0.70, 95% CI 0.53 to 0.94, I^2^=24%) and among ART-naïve patients (RR 0.78, 95% CI 0.65 to 0.94, I^2^=3%) but increased in African populations (RR 2.99, 95% CI 2.53 to 3.54, I^2^=0%).

In conclusion, exposure to INSTIs was not associated with increased risk of DM, except in the African population. Stratified analyses suggested reduced risk among ART-naïve patients and studies with longer follow-up.

International Prospective Register of Systematic Reviews (PROSPERO) registration number: CRD42021273040.

**What is already known on this topic**

- People living with HIV (PLHIV) have a higher prevalence of metabolic perturbations compared with HIV-negative populations, and integrase strand transfer inhibitors (INSTIs) are currently the preferred first-line and second-line antiretroviral therapy (ART).
- Some studies suggested more weight gain among INSTIs users compared with other ART regimens, while others reported accelerated hyperglycemia preceded by weight loss, weeks to a few months after initiating INSTIs.

**What this study adds**

- This systematic review and meta-analysis comprising ~75 000 PLHIV on different ART regimens is the first to examine the risk of insulin resistance and type 2 diabetes mellitus (DM) in INSTIs compared to other ART regimens.
- Analyses showed that compared to protease inhibitors and non-nucleoside reverse transcriptase inhibitors, INSTI exposure was not associated with increased risk of insulin resistance and/or DM.
- We also identified in multiple analyses that INSTIs might be associated with a reduced risk of type 2 DM in certain subpopulations of PLHIV.

**How this study might affect research, practice or policy**

- Our findings contribute to the evidence of metabolic safety of INSTI therapy, which might implicate the choice of therapy for millions of PLHIV.
- We demonstrated that exposure to INSTI therapy did not pose higher risk of insulin resistance and/or DM compared to other ART regimens. Initiating or switching to INSTIs is safe; nevertheless, monitoring is warranted in certain high-risk groups.

## Introduction

Antiretroviral therapy (ART) has revolutionized HIV treatment and significantly reduced AIDS-associated mortality globally, particularly in sub-Saharan Africa.^1^ People living with HIV (PLHIV) have more prevalent insulin resistance and diabetes mellitus (DM) than HIV-negative populations due to a combination of demographic and socioeconomic factors, in addition to HIV-related factors.^2 3^ HIV-associated chronic inflammation and certain forms of ART impair insulin signaling at target organs as well as insulin secretion.^4–6^ It remains challenging to distinguish to which extent the increased risk of DM is related to the normal aging process, the HIV infection, ART, or a combination of these factors.^7–12^

In the early ART era, nucleoside reverse transcriptase inhibitors (NRTIs) were coupled in combinations of predominantly stavudine, didanosine, zidovudine, lamivudine and zalcitabine with non-nucleoside reverse transcriptase inhibitors (NNRTIs).^13^ These drug combinations were linked to a spectrum of metabolic perturbations, including dyslipidaemia, lipodystrophy, and metabolic syndrome, and hence have largely been phased out of use.^14 15^ Since then, ART has conventionally included NNRTIs, protease inhibitors (PIs), and lately, the preferred integrase strand transfer inhibitors (INSTIs) as anchor agents coupled with largely metabolically safe NRTIs.^13 16 17^

In 2018, the WHO recommended the use of INSTIs, particularly dolutegravir (DTG) as first-line ART, and since then, the use of INSTIs has largely overtaken NNRTIs and PIs.^18^ This was after multiple countries reported primary resistance to NNRTIs above the recommended threshold of 10%.^19 20^ Thereafter, multiple studies demonstrated enhanced efficacy, a higher genetic barrier to resistance, good side effect profiles, and less drug–drug interactions with newer-generation integrase inhibitors.^21–25^ Despite their favorable side-effect profiles, INSTIs have consistently been associated with weight gain.^23 26^ Whether the weight gain in PLHIV translates to disorders in glucose metabolism in the long term remains to be demonstrated.^27^

Multiple case series on ART-experienced patients presenting with diabetic ketoacidosis with preceding weight loss a few weeks to months after starting INSTIs have been published.^24 25 28 29^ However, large population cohort studies have yielded conflicting results about the risk of diabetes among INSTI users.^30^

Given the inconsistent literature and to better quantify the risk, we performed a comprehensive literature review and meta-analyses aiming to summarize the current evidence on the association of INSTI therapy with insulin resistance, hyperglycemia, and incident DM versus PIs and NNRTI-based ART. We also explored the effect of other HIV-related factors and potential confounders on this association.

## Research design and methods

The protocol for this systematic review is registered on International Prospective Register of Systematic Reviews database (CRD42021273040) and published.^31^ This study is being reported according to the Preferred Reporting Items for Systematic Reviews and Meta-Analyses checklist.^32^ The link to the study dataset is listed in the online supplemental material (SD).

10.1136/bmjdrc-2022-003136.supp1 Supplementary data

### Search strategy and selection criteria

We searched PubMed/MEDLINE, Embase, and Web of Science (Clarivate) databases without language or geographical restrictions for randomized controlled trials (RCTs), cohort studies and case–control studies for eligible studies (online supplemental material, Emethods 1). Additionally, we searched Cochrane and clinicaltrials.org registries for eligible RCTs. Our search limit was fixed to the year 2000 to capture phase III clinical trial safety data, given that the first INSTIs, raltegravir, was approved by the Food and Drug Administration in 2007, and the search was last updated on 15 June 2022. We also searched abstracts of HIV conference meetings (International AIDS Society’s Conference on Retroviruses and Opportunistic Infections) for the same themes seeking studies that were eventually published. To identify relevant publications, two authors (FM and HK) independently screened all potential abstracts and reference lists in review articles. For published studies with desired outcomes but without data to calculate relative risk (RR) of diabetes, we reached out to authors for raw data. Studies eligible for full review were agreed on through consensus. A senior investigator (NB) was referred to in case of disagreement between the authors.

Studies were eligible if they reported risk of incident diabetes (or reported the required data to calculate incidence) with or without metabolic syndrome and/or insulin resistance, had exposure to INSTIs for ≥12 weeks, and had comparative arms of either NNRTI or PI anchored ART. Studies with cross-sectional design and studies including pregnant or breastfeeding mothers were excluded. Since we aimed to compare INSTIs versus PIs and/or NNRTIs as anchor agents, we also excluded studies where INSTIs were administered with PIs or NNRTIs in the same regimen. For studies with multiple publications, we included the publication with the most extended follow-up.

### Data analysis

We evaluated two outcomes: incident hyperglycemia and type 2 DM (new cases) as a discrete outcome or as part of metabolic syndrome (online supplemental table S1). A separate analysis was performed for mean changes in insulin resistance measured by the Homeostatic Model for Insulin Resistance (HOMA-IR) index, a factor of fasting blood glucose and insulin. We extracted variable study and population characteristics into excel forms (online supplemental table S3). Adjusted effect estimates were sought whenever reported; otherwise, raw data were retrieved.

The quality of the studies was assessed using the Newcastle-Ottawa Scale for cohort or case–control studies^33^ and the Revised Cochrane Risk-of-Bias tool for randomized trials^34^ (online supplemental tables S4 and S5).

Statistical analysis was done using meta-R package V.4.0.5 with R package Metaphor and Stata V.15 to generate forest plots of pooled effects with 95% CIs. We performed a random-effects meta-analysis adjusting for in-between-study heterogeneity to pool the risk (new cases/overall population at risk) of DM with or without metabolic syndrome (as discrete outcomes). The populations of interest were HIV patients exposed to INSTIs compared with patients on NNRTI or PI-based ART regimens. We assessed in-between study heterogeneity using the I^2^ statistic with DerSimonian and Laird’s method, using values <50%, 51%–74%, and ≥75% to represent low, moderate, and high heterogeneity, respectively.^35^ We sought evidence for publication bias by applying Egger’s test and visually inspecting funnel plots for asymmetry (if ≥10 studies).^36^ We also performed several subgroup analyses to explore if the risk of DM was affected by longevity on INSTIs, particular types of INSTIs, geographical region of study participants, and past exposure to ART, as some ART drugs were associated with abnormal glucose metabolism.^14^ To further explore sources of heterogeneity, we also carried out subanalyses by study design, type of caring facility, and type of non-INSTIs in the control group to compare pooled effects and heterogeneity. A p value of <0.1 was considered a statistically significant subgroup effect. We considered sensitivity analyses to test the robustness of our findings by including only studies reporting adjusted risk estimates, excluding studies with comorbidities like viral hepatitis B and C, studies where primary outcome was a metabolic endpoint and studies with no apparent conflict of interest. Studies reporting changes in mean HOMA-IR were separately analyzed to pool mean changes (on a continuous scale) of HOMA-IR pre-INSTI and post-INSTIs exposure compared with PIs and/or NNRTIs. Additionally, we performed a univariable metaregression to explore the effect of the following variables on the outcome: the effect of year of publication, follow-up duration, average age, CD4 count, body mass index (BMI) of participants and the proportion of male participants if at least 10 studies reported sufficient data. In this systematic review and meta-analysis, sex was defined as biological sex at birth.

## Results

### Literature search and study selection

**Figure 1:**
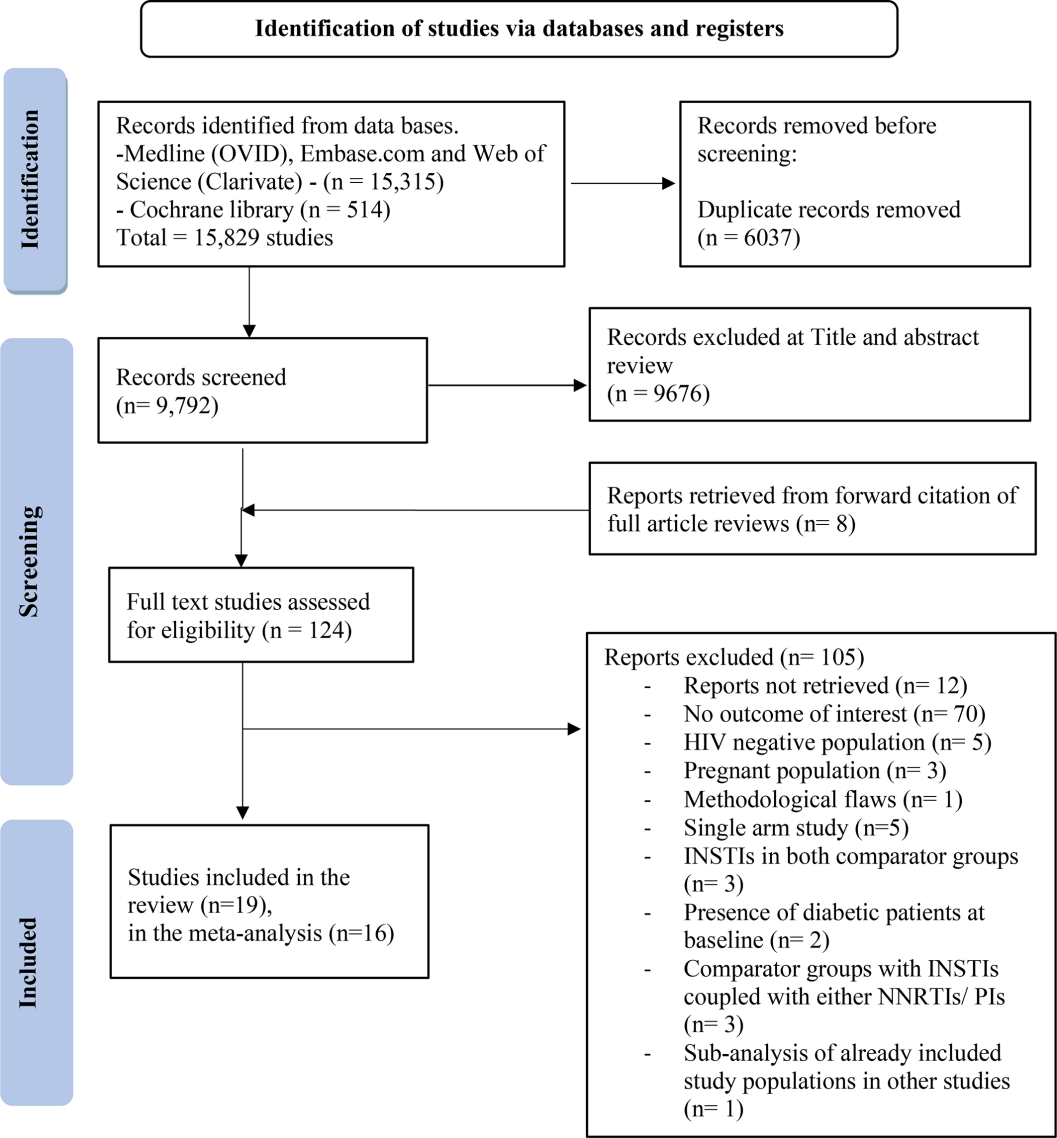
PRISMA flowchart for study selection. INSTI, integrase strand transfer inhibitor; NNRTI, non-nucleoside reverse transcriptase inhibitor; PI, protease inhibitor; PRISMA, Preferred Reporting Items for Systematic Reviews and Meta-Analyses.

Out of the 124 studies identified for full-text review, 16 studies were deemed eligible for inclusion in the meta-analysis,^27 30 37–50^ and 3 studies included in the systematic review could not be pooled in the quantitative synthesis^51–53^ (figure 1). Excluded studies and reasons for exclusion are presented in online supplemental table S6.

### Study characteristics

The 19 studies included in the systematic review (n=74 827) included 13 RCTs^37–42 45 46 48 49 51–53^ and 6 cohort studies^27 30 43 44 47 50^ (table 1). In the meta-analysis for incident DM, 13 of these studies, including 8 RCTs^37–42 45 46 48 49 51–53^ and 5 cohort studies,^27 30 43 44 47 50^ with a patient population of 72 404 were included. To analyze for the effect of INSTIs on insulin resistance, three studies including two RCTs^37–42 45 46 48 49 51–53^ and one cohort^27 30 43 44 47 50^ study with a patient population of 766 were pooled. Publications spanned from 2010^51^ to 2022,^50^ with patients’ enrolment from 2007 to 2018. Studies included cohorts from North America (six studies),^30 37 41 42 47 50^ Europe (five studies),^27 43 44 52 53^ Africa (two studies),^46 48^ and multinational (six studies).^38–40 45 47 51^ No studies originated from Asia.

**Table 1:** Study characteristics of the 13 studies included in the meta-analyses for incident type 2 DM and the three studies for insulin resistance

Study name	First author, journal, year of publication	Study design, setting	Area of origin of study participants (ART status at enrollment)	Outcome measure	On INSTIs (n)*	On non-INSTIs regimen (n)*	Duration of follow-up	Definition of DM	Definition of insulin resistance	Reported potential conflict of interest
STARTMRK trial	Rockstroh *et al*,^38^ *J Acquir Immune Defic Syndr*, 2013	RCT, 67 sites in five continents	Europe/Australia, North America, Latin America and South East Asia (ART-naïve)	RR	281	282	240 weeks	≥Grade 2 fasting hyperglycemia*	N/A	Yes
	Gupta *et al*,^37^ *J Acquir Immune Defic Syndr*, 2013	RCT, single center	USA (ART-experienced)	Mean changes in HOMA-IR	15	15	48 weeks	N/A	HOMA-IR	Yes
ACTG study A5257	Lennox *et al*,^41^ *Ann Intern Med*, 2014	RCT, multicenter	USA, Puerto Rico (ART-naïve)	RR	603	1208	96 weeks	≥Grade 2 fasting hyperglycemia*	N/A	None for the current study
FLAMINGO	Clotet *et al*,^39^ *Lancet*, 2014	RCT, 64 research centers	France, Germany, Italy, Puerto Rico, Romania, Russia, Spain, Switzerland and the USA (ART-naïve)	RR	242	242	96 weeks	≥Grade 2 fasting hyperglycemia*	N/A	Yes
SINGLE trial	Walmsley *et al*,^40^ *NEJM*, 2015	RCT, multicenter	North America, Europe and Australia (ART-naïve)	RR	414	419	48 weeks	≥Grade 2 fasting hyperglycemia*	N/A	Yes
	Dirajlal-Fargo *et al*,^42^ *Open Forum Infect Dis*, 2016	RCT, multicenter	USA (ART-naïve)	Mean changes in HOMA-IR	106	222	96 weeks	N/A	HOMA-IR	Yes
	Spagnuolo *et al*,^44^ *BMC Infectious Diseases*, 2017	Cohort, single center	Italy (Mixed population)	RR	772	5423	462 weeks	Two consecutive FPG ≥126 mg/dL or a 2-hour OGTT plasma glucose level ≥200 mg/dL or two consecutive fasting HbA1c levels of ≥48 mmol/mol, or a prescription for any antidiabetic medication	N/A	None for the current study
ANRS 12313 trial	Delaporte *et al*,^46^ (NAMSAL study group), *NEJM*, 2019	RCT, multicenter	Cameroon (ART-naïve)	RR	310	303	48 weeks	≥Grade 2 fasting hyperglycemia*	N/A	None for the current study
	Gianotti *et al*,^43^ *J Med Vir*, 2019	Cohort, single center	Italy (ART-naïve)	Mean changes in HOMA-IR	218	190 NNRTI, 210 PI/R	48 weeks	N/A	HOMA-IR	Yes
	Ursenbach *et al*,^27^ *J Antimicrob Chemother*, 2020	Cohort, multicenter in France and overseas	France (ART-naïve)	RR	3403	16 059	Variable	Documentation of diabetes in medical record, HbA1c >7.5%, being on DM treatment	N/A	None for the current study
	Rebeiro *et al*,^30^ *Clin Infect Dis*, 2020	Cohort, multicenter in North America	USA and Canada (ART-naïve)	RR	5183	17 701	Variable	HbA1c ≥6.5%, initiation of diabetes-specific medication or new DM diagnosis	N/A	Yes
ADVANCE trial	Venter *et al*,^48^ *Lancet HIV*, 2020	RCT, 11 public health clinics	South Africa (ART-naïve)	RR	690	347	96 weeks	Not stated	N/A	Yes
INSPIRING study	Dooley *et al*,^45^ *Clin Infect Dis*, 2020	RCT, multicenter	Argentina, Brazil, Mexico, Peru, Russia, South Africa, and Thailand (ART-naïve)	RR	69	44	52 weeks	≥Grade 2 fasting hyperglycemia*	N/A	Yes
	Hsu *et al*,^47^ *AIDS*, 2021	Cohort, 84 multicenter	USA (ART-naïve and experienced)	RR	15 122	2076	Variable	Recorded diagnosis of type 2 DM, antidiabetic medication prescription, lab tests indicative of DM	N/A	Yes
TANGO study	van Wyk *et al*,^49^ *J Acquir Immune Defic Syndr*, 2021	RCT, 134 multicenter in 10 countries	USA, Australia, Europe (ART-experienced)	RR	303	290	48 weeks	N/A	N/A	Yes

*The numbers represent patients without DM at baseline enrolled in the metabolic analyses in each study. NB Eron *et al*^51^ not included in the metanalyses.

†

ACTG, AIDS Clinical Trials Group; ART, antiretroviral therapy; DM, diabetes mellitus; FPG, Fasting Plasma Glucose; HbA1c, Glycated Hemoglobin; HOMA-IR, Homeostatic model of Insulin Resistance; INSTI, integrase strand transfer inhibitor; N/A, not available; OGTT, Oral Glucose Tolerance Test; RCT, randomized controlled trial; RR, relative risk.

The majority of studies (n=15)^27 30 38 39 41 42 45–53^ involved multiple centers, while four studies were single centers.^37 43 44 50^ Eight studies^38–41 45 46 48 49^ reported virological primary outcomes, mentioning hyperglycemia among the safety data, while 11 studies had metabolic endpoints.^27 30 37 42–44 47 50–53^ In 14 studies,^27 30 38–41 43–49 51^ crude numbers of DM were retrieved, while 5 studies^37 42 43 52 53^ reported mean changes in HOMA-IR. Four studies^27 30 47 50^ provided adjusted estimates for incidence of DM, 4 studies^46–48 50^ reported weight changes with INSTI exposure. None of the studies reported DM as part of metabolic syndrome as an outcome.

Overall, the quality of the studies was rated as high (online supplemental table S4 and S5). Common to most RCTs was a lack of blinding in the assessment of the outcome.

### Study population characteristics

A total of 74 827 participants were included in the systematic review. The sample size ranged from 30^37^ to 22 884^30^ patients. Overall, 37.8% (n=28 289) of patients used INSTIs, particularly Elvitegravir (n=10 218), DTG (n=9783) and raltegravir (n=4478).

Non-INSTI users constituted 62.2% (n=46 538) with 21 391 receiving PIs^27 30 39 41 47 51 54^ and 17 842 receiving NNRTIs.^27 30 38 40 45 46 48^ The mean follow-up duration was 21.2 months, ranging from 5.6^37^ to 108.0 months.^44^ In INSTI populations, the mean age was 38.7 (IQR 27–54) years, similar to 38.4 (IQR 27.0–54.6) years in non-INSTI populations. Two studies included populations <18 years.^47 48^

In the INSTI group, 82.1% (n=23 231) was male, contrasted to 68.8% (n=32 037) in non-INSTI groups. One study enrolled only female participants.^53^

All studies reported HIV RNA levels at baseline, with 12 studies^27 30 38–43 45 46 48 50^ enrolling ART-naïve participants, 5 studies^37 49 51–53^ enrolling ART-experienced patients and 2 studies enrolling both ART-naïve and ART-experienced patients.^27 30 39 41 47 51 54^

### Risk of incident DM and hyperglycemia with exposure to INSTIs

**Figure 2:**
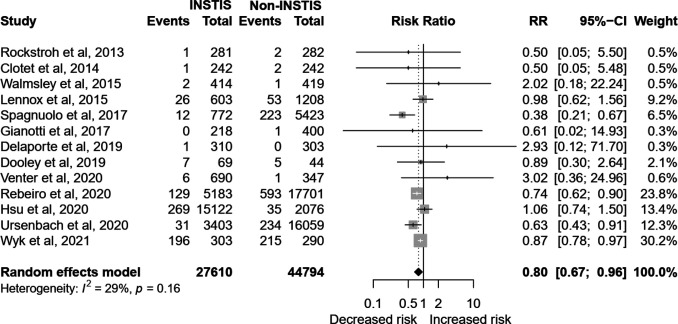
Forest plot of the association of INSTI exposure to incident hyperglycemia and diabetes mellitus compared with other art regimens. The crude numbers of events are based on the longest follow-up reported in the studies. INSTI, integrase strand transfer inhibitor; RR, relative risk.

In the 13 pooled studies^27 30 38–41 43–49^ (n=72 404), INSTI exposure carried a lower risk of incident DM as compared with any other ART (n=13, RR 0.80, 95% CI 0.67 to 0.96, I^2^=29%; figure 2). Particularly the risk was lower when compared with NNRTIs^27 30 38 40 45 46 48^ (n=7, RR 0.75, 95% CI 0.63 to 0.89, I^2^=0%) and borderline when compared with PIs^27 30 39 41 43 47^ (n=6, RR 0.78, 95% CI 0.61 to 1.01, I^2^=27%). There was minimal heterogeneity in both the aforementioned subanalyses (online supplemental figure S1). The test for subgroup difference indicated no statistically significant subgroup effect (p=0.74), suggesting that use of either PI or NNRTIS did not modify the lower risk in INSTIs group.

Additionally, the risk reduction of diabetes was more evident in studies with a longer follow-up (≥12 months) (n=8, RR 0.70 95% CI 0.53 to 0.94, I^2^=24%)^27 30 38 41 43–45 48^ (online supplemental figure S2), than studies with less than 1 year follow-up (n=6, RR 0.89 95% CI 0.80 to 0.99, I^2^=0). The test for subgroup difference was significant (p=0.07), suggesting that longer follow-up influenced INSTI association with the outcome. The association between INSTIs and lower risk of DM was demonstrated in studies enrolling only adults (RR 0.77, 95% CI 0.65 to 0.91, I^2^=26%)^27 30 38–41 43–46 49^ and in multicenter studies (RR 0.84, 95% CI 0.77 to 0.93, I^2^=0%).^27 30 38–41 45–49^ INSTI use in PLHIV of African origin was associated with a threefold increased risk of DM in two studies with minimal heterogeneity (RR 2.99, 95% CI 2.53 to 3.54, I^2^=0%),^46 48^ and a significant subgroup effect was demonstrated by area of origin (p<0.01). Further interpretation of subgroup analyses is reported in table 2.

**Table 2:** Subanalysis for the risk of diabetes mellitus with exposure to INSTIs in people living with HIV

Analysis	Arms	Studies (n)	References	INSTIs group (n)	Non-INSTIs group (n)	RR (95% CI)	Heterogeneity (I^2^), P value	Subgroup analysis: P value, heterogeneity (I^2^)	Interpretation of subgroup analysis
All studies	INSTIs versus PI and/or NNRTIs	13	27 30 38–41 43–49	27 610	44 794	**0.80 (0.67 to 0.96)**	29%	–	–
INSTI versus other drug classes	INSTI versus PI	6	27 30 39 41 43 47	24 771	21 049	0.78 (0.61 to 1.01)	27%	0.74, 0%	No subgroup effect, minimal heterogeneity
	INSTI versus NNRTI	7	27 30 38 40 45 46 48	10 350	17 842	**0.75 (0.63 to 0.89)**	0%		
By ART status at baseline	ART-naïve	11	27 30 38–41 43–46 48	17 940	37 972	**0.78 (0.65 to 0.94)**	3%	0.29, 23%	No subgroup effect, minimal heterogeneity
	ART-experienced	2	47 49	8900	1389	0.85 (0.43 to 1.68)	0%		
By presence of conflict of interest	Reported conflict of interest	9	30 38–40 43 45 47–49	22 522	21 801	**0.85 (0.78 to 0.93)**	0%	0.44, 23%	No subgroup effect, minimal heterogeneity
	No reported conflict of interest	4	27 41 44 46	5 088	22 993	0.65 (0.29 to 1.46)	59%		
By age	Participants ≥18 years	11	27 30 38–41 43–46 49	11 798	42 371	**0.77 (0.65 to 0.91)**	26%	0.07, 29%	Statistically significant, qualitative subgroup effect
	Included participants below 18 years	2	47 48	15 812	2423	1.09 (0.13 to 9.29)	0%		
By study setting	Multicenter	11	27 30 38–41 45–49	26 620	38 971	**0.84 (0.77 to 0.93**)	0%	<0.01, 23%	Statistically significant, quantitative subgroup effect
	Single center	2	43 44	990	5823	0.38 (0.13 to 1.11)	0%		
By geographical origin of the study participants	Multinational	5	38–40 45 47	1309	1277	**0.87 (0.81 to 0.94**)	0%	<0.01, 13%	Statistically significant subgroup effect by region of origin, qualitative effect
	African	2	46 48	1000	650	2.99 (2.53 to 3.54)	0%		
	North American	3	30 41 47	20 908	20 985	0.86 (0.53 to 1.42)	45%		
	Europe	3	27 43 44	4393	21 882	0.54 (0.27 to 1.08)	3%		
By study design	RCT	8	38–41 45 48 49	2912	3135	**0.88 (0.81 to 0.96**)	0%	0.27, 12%	No subgroup effect, minimal heterogeneity
	Cohort	5	27 30 43 44 47	24 698	41 659	0.69 (0.44 to 1.10)	60%		
By primary outcome	Virological outcome	8	38–41 45 46 48 49	2912	3135	**0.88 (0.81 to 0.96**)	0%	0.16, 29%	No subgroup effect. Moderate heterogeneity.
	Metabolic outcome	5	27 30 43 44 47	24 698	41 659	0.69 (0.44 to 1.10)	60%		
By follow-up duration	≥12 months	8	27 30 38 41 43–45 48	11 219	41 464	**0.70 (0.53 to 0.94**)	24%	0.07, 29%	No subgroup effect, minimal heterogeneity
	<12 months	6	39 40 45–47 49	16 460	3374	**0.88 (0.79 to 0.99**)	0%		
By type of INSTI in ART-naïve patients	Dolutegravir	7	30 39 40 45–48	5751	17 679	0.94 (0.53 to 1.67)	43%	0.35, 61%	No subgroup effect, moderate heterogeneity
	Elvitegravir	2	30 47	5819	16 324	0.80 (0.01 to 123.82)	78%		
	Raltegravir	4	30 38 41 47	2172	17 814	1.23 (0.91 to 1.66)	0%		
By type of INSTI in ART-experienced patients	Dolutegravir	2	47 49	3889	1389	0.92 (0.21 to 3.99)	0%	0.57, 0%	No subgroup effect, minimal heterogeneity
	Elvitegravir	1	47	4281	1109	0.75 (0.48 to 1.17)	–		
	Raltegravir	1	47	730	1109	1.09 (0.60 to 1.99)	–		
By viral hepatitis comorbidities	Hepatitis B and C included	10	27 30 38–41 43–49	26 824	44 041	0.76 (0.58 to 1.00)	67.2%	0.88, 12%	No subgroup effect, minimal heterogeneity
	Only hepatitis C included	3	27 30 38–41 43–49	786	753	0.87 (0.78 to 0.98)	32.8%		
By studies providing adjusted risk estimates		5				0.83 (0.58 to 1.18)	100%	–	–

the boldfaced values are Statistically significant.

.ART, antiretroviral therapy; INSTI, integrase strand transfer inhibitor; NNRTI, non-nucleoside reverse transcriptase inhibitor; PI, protease inhibitor; RCT, randomized controlled trial; RR, relative risk.

### Risk of DM and hyperglycemia in treatment naïve or experienced individuals

The risk of DM on exposure to INSTIs was reduced in ART-naïve patients (n=11, RR 0.78, 95% CI 0.65 to 0.94, I^2^=3%)^27 30 38–41 43–46 48^ but equal to controls in ART-exposed patients (n=2, RR 0.85, 95% CI 0.43 to 1.62, I^2^=0%)^47 49^ (online supplemental figure S4 and table 2).

No differences in the risk of DM were noted in studies per individual types of INSTIs in ART-naïve patients: DTG (n=7, RR 0.94, 95% CI 0.53 to 1.67, I^2^=43%),^30 39 40 45–48^ elvitegravir (n=2, RR 0.80, 95% CI 0.01 to 123.82, I^2^=78%)^30 47^ and raltegravir (n=4, RR 1.23, 95% CI 0.91 to 1.6, I^2^=0%).^30 38 41 47^

The risk of DM was lower in five cohort studies providing adjusted estimates, although not statistically significant (n=5, RR 0.83, 95% CI 0.58 to 1.18).^27 30 47 49 50^ In RCTs, the risk of developing hyperglycemia and/or DM was lower (n=8, RR 0.88, 95% CI 0.81 to 0.96) with minimal heterogeneity (I^2^=0%).^38–41 45 48 49^ A trend toward decreased risk was also observed in cohort studies, yet not statistically significant (n=5, RR 0.69, 95% CI 0.44 to 1.10) and with substantial heterogeneity (I^2^=60%)^27 30 43 44 47^ (online supplemental figure S5).

### Effect of weight gain

We sought to analyze the effect of baseline weight, weight gain or changes in BMI on the incidence of diabetes in the study populations. Eleven studies^27 30 38–41 43–49^ provided estimates of weight and/ or BMI at baseline, yet changes were not presented per type of ARTs nor stratified per persons who developed diabetes and/or hyperglycemia, making it difficult to analyze.

### Effect of exposure to INSTIs on insulin resistance

In the three included studies (n=976), INSTIs were associated with an insignificant increase in mean HOMA-IR from baseline compared with non-INSTIs (0.78, 95% CI −0.15 to 1.70) with substantial heterogeneity (I^2^=82.5%).^37 42 43^ The same results were noted when comparing INSTIs to PIs (0.90, 95% CI −0.90 to 2.69) and to NNRTIs (0.17, 95% CI −0.44 to 0.79) with substantial heterogeneity in both analyses (online supplemental figure S7).

For studies reporting incident insulin resistance and/or diabetes across different meta-analyses when the number of studies was ≥10, no publication bias or small study effect was detected by funnel plot asymmetry and by Egger’s test (online supplemental figure S6).

### Metaregression analysis

We further explored the influence of specific study and HIV-related factors on the pooled risk of developing insulin resistance and/or type 2 DM between INSTIs and non-INSTI comparators. Neither the proportions of male, black population, or publication year were associated with the pooled risk in univariable meta-regression analysis. However, studies with longer follow-up duration were significantly associated with lower risk of type 2 DM in INSTIs compared with non-INSTIs (online supplemental table S7 and figure S9).

### Influence analysis

We conducted influence analysis by the leave-one-out method to investigate the individual impact of each study (online supplemental figure S10). There was no significant change in the pooled effect estimates. Baujat plot pointed to one study with the most impact on overall study heterogeneity yet with minimal effect on the pooled effect estimates (online supplemental Figure S11).

## Discussion

In this comprehensive systematic review and meta-analysis of approximately 75 000 PLHIV exposed to different ART regimens, INSTI use was associated with a lower risk of incident DM and hyperglycemia compared with NNRTI and PI anchored ART. Particularly, ART-naïve PLHIV and prolonged follow-up studies suggested a lower risk of DM among the INSTI group compared with the non-INSTI group. The association was consistent when pooling eight RCTs in the analysis. By contrast, PLHIV of African origin treated with INSTIs had a threefold increased risk of DM compared with their non-INSTI peers. Analysis per individual type of INSTIs showed similar risk compared with peer non-INSTIs, with raltegravir demonstrating a trend toward a higher risk compared with elvitegravir and DTG. Univariable regression analysis suggested that studies with longer follow-up times showed a lower risk.

Multiple cases of accelerated hyperglycemia in patients starting INSTIs have been reported, particularly on DTG and, in a few cases, raltegravir.^25 55^ The common presentation was diabetic ketoacidosis preceded by weight loss, weeks to months after initiating therapy, which might represent a typical phenotype of insulin deficiency.^25 29^ Some of the postulated mechanisms for the accelerated hyperglycemia included intracellular magnesium chelation induced by INSTIs leading to altered hepatic and skeletal muscle insulin signaling, mitochondrial dysfunction from previous exposure to more toxic NRTIs and possible genetic predisposition (online supplemental file 1, ref. 57).^56^ Interestingly, at the population level, INSTIs particularly DTG have been consistently associated with weight gain (online supplemental file 1, ref. 58). A recent systematic review concluded that INSTIs have a higher risk of DM compared with alternative backbone ART regimens.^56^ Most of the conclusions in that narrative review were premised on the consistent association of INSTIs with weight gain, a known precursor for metabolic syndrome or DM.^56^ We could not conclusively ascertain the effects of weight gain on the incidence of diabetes as data were lacking to perform a subanalysis for BMI changes. In the current analysis, studies with follow-up more than 12 months showed a 30% lower risk of type 2 DM among INSTIs versus non-INSTIs. This reduced risk tended to attenuate when restricted to studies with shorter follow-up. We observed a trend toward more insulin resistance (increase in HOMA-IR) rather than overt type 2 DM among INSTIs compared with non-INSTIs (online supplemental figure S7). It is unclear whether this trend is induced by the increased weight accompanying the ‘return-to-health phenomenon’ with possible metabolic perturbations in some susceptible individuals or could lead to overt type 2 DM and metabolic syndrome in the long term (online supplemental file 1, ref. 59). Considering the small sample size of this analysis (three studies with 766 patients) and the heterogeneity of PLHIV populations, long-term follow-up studies are therefore warranted, particularly accounting for sex, the presence of malnutrition, obesity, and/or metabolic syndrome at treatment initiation.

A threefold increased risk of diabetes in African patients was observed in the subanalysis by geographical origin. These two pooled studies^46 48^ were high-quality RCTs involving ART-naïve adults with primarily virological outcomes. They included ART-naïve patients of mean baseline age 32–38 years with unsuppressed viral loads and mean baseline CD4s of 280 and 336 cells/mm^3^. The baseline BMI for both studies did not significantly differ from the mean BMI from other meta-analyzed studies with BMI data. Exposure groups had patients on DTG and comparator groups, efavirenz. Estimates of metabolic syndrome prevalence among PLHIV in SSA range from 13% to 58%, with a higher proportion among ART-experienced than among ART-naïve (online supplemental file 1, ref. 60). It is likely that the increased risk of type 2 DM observed is driven by the higher prevalence of metabolic syndrome in this population (online supplemental file 1, ref. 60). On metaregression for age, baseline CD4, and viral load, we found a pattern of an increased risk of diabetes with higher baseline viral loads and low CD4 cell counts (online supplemental file 1, figure S8). This is in tandem with the known literature suggesting that chronically heightened inflammation in patients with high viral loads is a driver of insulin resistance and hence a precursor of type 2 DM (online supplemental file 1, ref. 61). These factors could have been drivers of this risk in this African population with more likely late presentation compared with PLHIV in resource-affluent settings. These results should, however, be interpreted with caution, given there were only two studies meta-analyzed with a small patient population; hence, these findings may not be extrapolated to the general African population. Studies suggested that women living with HIV have higher risk of ART-related weight gain compared with men (online supplemental file 1, ref. 62); moreover, women with HIV have higher odds of type 2 DM compared with women without HIV infection(online supplemental file 1, ref. 63). This might be attributed to higher weight gain, and possibly more prevalent cardiometabolic risk factors in women population with HIV. Whether African women living with HIV have heightened risk for type 2 DM compared with male peers is debated. In a meta-analysis of 20 studies from Africa, the prevalence of type 2 DM was similar in HIV and non-HIV populations regardless of sex, and similar prevalence was noted between treated and untreated PLHIV, though in between-studies heterogeneity was high (online supplemental file 1, ref. 64). In our analysis, sex was not associated with the pooled risk of type 2 DM in metaregression analysis.

INSTI exposure was associated with a low risk of diabetes, noted in ART-naïve populations compared with ART-experienced patients. This is in line with collection of reports on lower prevalence of metabolic syndrome in ART-naïve versus ART-experienced patients (online supplemental file 1, ref. 61). Another potential explanation might be thatbe clinicians tended not to start INSTIs in patients at high risk of diabetes, which could not be applied to ART-exposed patients being switched to INSTIs due to virological failure with less consideration for metabolic risk (online supplemental file 1, ref. 65 and 66).

We encountered certain limitations such as insufficient data on possible factors affecting glucose metabolism, which are potential confounders such as changes in BMI, family history of diabetes, lifestyle, concurrent drugs such as steroids and gender-affirming hormonal therapy in transgender patients. In the ART-experienced populations, we could not adjust for prior exposure to drugs like stavudine, didanosine, and zidovudine, known to cause lipodystrophy, insulin resistance and dyslipidaemia due to lack of patient-level data. There was variation in the criteria used to define diabetes in the different studies, with most retrospective cohort studies using multiple criteria: HBA1C, fasting blood glucose, oral glucose tolerance tests, and prescriptions for diabetes medication, while most RCTs used division of AIDS grading of fasting blood glucose (online supplemental file 1, ref. 67). To partially account for these limitations, we conducted influence and stratified analyses by study design, primary metabolic outcome, and ART status. There was minimal heterogeneity and an absence of publication bias across several subgroups and sensitivity analyses.

## Conclusion

In conclusion, this meta-analysis demonstrated that INSTI use was not associated with an increased risk of DM compared with PIs and NNRTIs except in African PLHIV. There is a need for long-term follow-up studies with primarily metabolic outcomes to ascertain these results further and delineate the contribution of weight gain in PLHIV exposed to INSTIs on glucose dysmetabolism. Additionally, the increased risk of DM in African PLHIV merits more targeted research as this population in the meta-analysis was largely under-represented.

## Data Availability

All data relevant to the study are included in the article or uploaded as supplementary information.

## References

[1] Global HIV & AIDS statistics — fact sheet | UNAIDS. Available: https://www.unaids.org/en/resources/fact-sheet [Accessed 28 Mar 2022].

[2] TREAT ALL: POLICY ADOPTION AND IMPLEMENTATION STATUS IN COUNTRIES HIV TREATMENT AND CARE. 2017.

[3] Unaids. n.d. Responding to the challenge of non-communicable diseases. 10.1097/QAD.0000000000001888

[4] Jespersen NA, Axelsen F, Dollerup J, et al. The burden of non-communicable diseases and mortality in people living with HIV (PLHIV) in the pre-, early- and late-HAART era. HIV Med 2021;22:478–90. 10.1111/hiv.13077 33645000 PMC8247855

[5] Kumar S, Samaras K. The impact of weight gain during HIV treatment on risk of pre-diabetes, diabetes mellitus, cardiovascular disease, and mortality. Front Endocrinol (Lausanne) 2018;9:705.:705. 10.3389/fendo.2018.00705 30542325 PMC6277792

[6] Koethe JR, Jenkins CA, Lau B, et al. Rising obesity prevalence and weight gain among adults starting antiretroviral therapy in the United States and Canada. AIDS Res Hum Retroviruses 2016;32:50–8. 10.1089/aid.2015.0147 26352511 PMC4692122

[7] Maggi P, Di Biagio A, Rusconi S, et al. Cardiovascular risk and dyslipidemia among persons living with HIV: a review. BMC Infect Dis 2017;17:551. 10.1186/s12879-017-2626-z 28793863 PMC5550957

[8] Florescu D, Kotler DP. Insulin resistance, glucose intolerance and diabetes mellitus in HIV-infected patients. Antiviral Therapy 2007;12:149–62. 10.1177/135965350701200214 Available: https://doi.org/101177/135965350701200214 17503657

[9] Pedro MN, Rocha GZ, Guadagnini D, et al. Insulin resistance in HIV-patients: causes and consequences. Front Endocrinol (Lausanne) 2018;9:514.:514. 10.3389/fendo.2018.00514 30233499 PMC6133958

[10] Hulgan T. Factors associated with insulin resistance in adults with HIV receiving contemporary antiretroviral therapy: a brief update. Curr HIV/AIDS Rep 2018;15:223–32. 10.1007/s11904-018-0399-7 29700760 PMC6003865

[11] Tseng A, Seet J, Phillips EJ. The evolution of three decades of antiretroviral therapy: challenges, triumphs and the promise of the future. Br J Clin Pharmacol 2015;79:182–94. 10.1111/bcp.12403 24730660 PMC4309625

[12] Pao V, Lee GA, Grunfeld C. HIV therapy, metabolic syndrome, and cardiovascular risk. Curr Atheroscler Rep 2008;10:61–70. 10.1007/s11883-008-0010-6 18366987 PMC3166347

[13] Ergin HE, Inga EE, Maung TZ, et al. HIV, antiretroviral therapy and metabolic alterations: A review. Cureus 2020;12:e8059. 10.7759/cureus.8059 32537277 PMC7286589

[14] Krishnan S, Schouten JT, Atkinson B, et al. Metabolic syndrome before and after initiation of antiretroviral therapy in treatment-naive HIV-infected individuals. J Acquir Immune Defic Syndr 2012;61:381–9. 10.1097/QAI.0b013e3182690e3c 22828718 PMC3480980

[15] Vella S, Schwartländer B, Sow SP, et al. The history of antiretroviral therapy and of its implementation in resource-limited areas of the world. AIDS 2012;26:1231–41. 10.1097/QAD.0b013e32835521a3 22706009

[16] Messiaen P, Wensing AMJ, Fun A, et al. Clinical use of HIV integrase inhibitors: a systematic review and meta-analysis. PLoS One 2013;8:e52562. 10.1371/journal.pone.0052562 23341902 PMC3541389

[17] Yoshinaga T, Miki S, Kawauchi-Miki S, et al. Barrier to resistance of dolutegravir in two-drug combinations. Antimicrob Agents Chemother 2019;63:e02104-18. 10.1128/AAC.02104-18 30602514 PMC6395897

[18] Stellbrink H-J, Reynes J, Lazzarin A, et al. Dolutegravir in antiretroviral-naive adults with HIV-1: 96-week results from a randomized dose-ranging study. AIDS 2013;27:1771–8. 10.1097/QAD.0b013e3283612419 23807273 PMC3694319

[19] Castagna A, Maggiolo F, Penco G, et al. Dolutegravir in antiretroviral-experienced patients with raltegravir- and/or elvitegravir-resistant HIV-1: 24-week results of the phase III VIKING-3 study. J Infect Dis 2014;210:354–62. 10.1093/infdis/jiu051 24446523 PMC4091579

[20] Llibre JM, Hung C-C, Brinson C, et al. Efficacy, safety, and tolerability of dolutegravir-rilpivirine for the maintenance of virological suppression in adults with HIV-1: phase 3, randomised, non-inferiority SWORD-1 and SWORD-2 studies. Lancet 2018;391:839–49.:S0140-6736(17)33095-7. 10.1016/S0140-6736(17)33095-7 29310899

[21] Trottier B, Lake JE, Logue K, et al. Dolutegravir/abacavir/lamivudine versus current ART in virally suppressed patients (STRIIVING): a 48-week, randomized, non-inferiority, open-label, phase iiib study. Antivir Ther 2017;22:295–305. 10.3851/IMP3166 28401876

[22] Kolakowska A, Maresca AF, Collins IJ, et al. Update on adverse effects of HIV integrase inhibitors. Curr Treat Options Infect Dis 2019;11:372–87. 10.1007/s40506-019-00203-7 33380904 PMC7758219

[23] Eckard AR, McComsey GA. Weight gain and integrase inhibitors. Curr Opin Infect Dis 2020;33:10–9. 10.1097/QCO.0000000000000616 31789693 PMC7433018

[24] Fong PS, Flynn DM, Evans CD, et al. Integrase strand transfer inhibitor-associated diabetes mellitus: A case report. Int J STD AIDS 2017;28:626–8. 10.1177/0956462416675107 27733708

[25] Lamorde M, Atwiine M, Owarwo NC, et al. Dolutegravir-associated hyperglycaemia in patients with HIV. Lancet HIV 2020;7:e461–2.:S2352-3018(20)30042-4. 10.1016/S2352-3018(20)30042-4 32105626

[26] Lake JE, Trevillyan J. Impact of integrase inhibitors and tenofovir alafenamide on weight gain in people with HIV. Curr Opin HIV AIDS 2021;16:148–51. 10.1097/COH.0000000000000680 33797433

[27] Ursenbach A, Max V, Maurel M, et al. Incidence of diabetes in HIV-infected patients treated with first-line integrase strand transfer inhibitors: a French multicentre retrospective study. J Antimicrob Chemother 2020;75:3344–8. 10.1093/jac/dkaa330 32791523

[28] Nolan NS, Adamson S, Reeds D, et al. Bictegravir-based antiretroviral therapy-associated accelerated hyperglycemia and diabetes mellitus. Open Forum Infect Dis 2021;8:ofab077. 10.1093/ofid/ofab077 33981777 PMC8103800

[29] McLaughlin M, Walsh S, Galvin S. Dolutegravir-induced hyperglycaemia in a patient living with HIV. J Antimicrob Chemother 2018;73:258–60. 10.1093/jac/dkx365 29077869

[30] Rebeiro PF, Jenkins CA, Bian A, et al. Risk of incident diabetes mellitus, weight gain, and their relationships with integrase inhibitor-based initial antiretroviral therapy among persons with human immunodeficiency virus in the united states and canada. Clin Infect Dis 2021;73:e2234–42. 10.1093/cid/ciaa1403 32936919 PMC8492142

[31] Mulindwa F, Kamal H, Castelnuovo B, et al. Association between integrase strand transfer inhibitor (instis) use with insulin resistance and incident diabetes mellitus in persons living with HIV: A systematic review and meta-analysis protocol. PLoS One 2022;17:e0264792. 10.1371/journal.pone.0264792 35235607 PMC8890726

[32] N.d. PRISMA 2020 checklist section and topic item # checklist item location where item is reported TITLE 1 identify the report as a systematic review. 10.1136/bmj.n71

[33] Ottawa hospital research institute. Available: http://www.ohri.ca/programs/clinical_epidemiology/oxford.asp [Accessed 28 Mar 2022].

[34] RoB 2: A revised cochrane risk-of-bias tool for randomized trials | cochrane bias. Available: https://methods.cochrane.org/bias/resources/rob-2-revised-cochrane-risk-bias-tool-randomized-trials [Accessed 28 Mar 2022].

[35] Cochrane handbook for systematic reviews of interventions | cochrane training. Available: https://training.cochrane.org/handbook/current [Accessed 28 Mar 2022].

[36] Egger M, Davey Smith G, Schneider M, et al. Bias in meta-analysis detected by a simple, graphical test. BMJ 1997;315:629–34. 10.1136/bmj.315.7109.629 9310563 PMC2127453

[37] Gupta SK, Mi D, Moe SM, et al. Effects of switching from efavirenz to raltegravir on endothelial function, bone mineral metabolism, inflammation, and renal function: a randomized, controlled trial. J Acquir Immune Defic Syndr 2013;64:279–83. 10.1097/qai.0b013e3182a97c39 24278992 PMC4091630

[38] Rockstroh JK, DeJesus E, Lennox JL, et al. Durable efficacy and safety of raltegravir versus efavirenz when combined with tenofovir/emtricitabine in treatment-naive HIV-1-infected patients: final 5-year results from STARTMRK. J Acquir Immune Defic Syndr 2013;63:77–85. 10.1097/QAI.0b013e31828ace69 23412015

[39] Clotet B, Feinberg J, van Lunzen J, et al. Once-daily dolutegravir versus darunavir plus ritonavir in antiretroviral-naive adults with HIV-1 infection (FLAMINGO): 48 week results from the randomised open-label phase 3b study. Lancet 2014;383:2222–31.:S0140-6736(14)60084-2. 10.1016/S0140-6736(14)60084-2 24698485

[40] Walmsley S, Baumgarten A, Berenguer J, et al. Brief report: dolutegravir plus abacavir/lamivudine for the treatment of HIV-1 infection in antiretroviral therapy-naive patients: week 96 and week 144 results from the SINGLE randomized clinical trial. J Acquir Immune Defic Syndr 2015;70:515–9. 10.1097/QAI.0000000000000790 Available: www.jaids.com 26262777 PMC4645960

[41] Lennox JL, Landovitz RJ, Ribaudo HJ, et al. A phase III comparative study of the efficacy and tolerability of three non-nucleoside reverse transcriptase inhibitor-sparing antiretroviral regimens for treatment-naïve HIV-1-infected volunteers: A randomized. Controlled Trial 2014;161:461–71. 10.7326/L15-5066-3 PMC4412467 25285539

[42] Dirajlal-Fargo S, Moser C, Brown TT, et al. Changes in insulin resistance after initiation of raltegravir or protease inhibitors with tenofovir-emtricitabine: AIDS clinical trials group a5260s. Open Forum Infect Dis 2016;3:ofw174. 10.1093/ofid/ofw174 27704026 PMC5047417

[43] Gianotti N, Muccini C, Galli L, et al. Homeostatic model assessment for insulin resistance index trajectories in HIV-infected patients treated with different first-line antiretroviral regimens. J Med Virol 2019;91:1937–43. 10.1002/jmv.25541 31286527

[44] Spagnuolo V, Galli L, Poli A, et al. Associations of statins and antiretroviral drugs with the onset of type 2 diabetes among HIV-1-infected patients. BMC Infect Dis 2017;17:1–10. 10.1186/s12879-016-2099-5 28061820 PMC5219726

[45] Dooley KE, Kaplan R, Mwelase N, et al. Dolutegravir-based antiretroviral therapy for patients coinfected with tuberculosis and human immunodeficiency virus: a multicenter, noncomparative, open-label, randomized trial. Clin Infect Dis 2020;70:549–56. 10.1093/cid/ciz256 30918967

[46] NAMSAL ANRS 12313 Study Group, Kouanfack C, Mpoudi-Etame M, et al. Dolutegravir-based or low-dose efavirenz-based regimen for the treatment of HIV-1. N Engl J Med 2019;381:816–26. 10.1056/NEJMoa1904340 31339676

[47] Hsu R, Brunet L, Fusco JS, et al. Incident type 2 diabetes mellitus after initiation of common HIV antiretroviral drugs. AIDS 2021;35:81–90. 10.1097/QAD.0000000000002718 33048874

[48] Venter WDF, Sokhela S, Simmons B, et al. Dolutegravir with emtricitabine and tenofovir alafenamide or tenofovir disoproxil fumarate versus efavirenz, emtricitabine, and tenofovir disoproxil fumarate for initial treatment of HIV-1 infection (advance): week 96 results from a randomised, phase 3, non-inferiority trial. Lancet HIV 2020;7:e666–76.:S2352-3018(20)30241-1. 10.1016/S2352-3018(20)30241-1 33010240

[49] van Wyk J, Ait-Khaled M, Santos J, et al. Brief report: improvement in metabolic health parameters at week 48 after switching from a tenofovir alafenamide-based 3- or 4-drug regimen to the 2-drug regimen of dolutegravir/lamivudine: the tango study. J Acquir Immune Defic Syndr 2021;87:794–800. 10.1097/QAI.0000000000002655 33587500 PMC8126488

[50] Asundi A, Olson A, Jiang W, et al. Integrase inhibitor use associated with weight gain in women and incident diabetes mellitus. AIDS Res Hum Retroviruses 2022;38:208–15. 10.1089/AID.2021.0091 Available: https://home.liebertpub.com/aid 2022;38:208–15 34877881 PMC8968841

[51] Eron JJ, Young B, Cooper DA, et al. Switch to a raltegravir-based regimen versus continuation of a lopinavir-ritonavir-based regimen in stable HIV-infected patients with suppressed viraemia (SWITCHMRK 1 and 2): two multicentre, double-blind, randomised controlled trials. Lancet 2010;375:396–407. 10.1016/S0140-6736(09)62041-9 20074791

[52] Saumoy M, Sánchez-Quesada JL, Martínez E, et al. Ldl subclasses and lipoprotein-phospholipase A2 activity in suppressed HIV-infected patients switching to raltegravir: spiral substudy. Atherosclerosis 2012;225:200–7.:S0021-9150(12)00557-6. 10.1016/j.atherosclerosis.2012.08.010 23017355

[53] Ibrahim F, Samarawickrama A, Hamzah L, et al. Bone mineral density, kidney function, weight gain and insulin resistance in women who switch from TDF/FTC/NNRTI to ABC/3TC/DTG. HIV Med 2021;22:83–91. 10.1111/hiv.12961 32985122

[54] Gianotti N, Poli A, Nozza S, et al. Durability of switch regimens based on rilpivirine or on integrase inhibitors, both in association with tenofovir and emtricitabine, in HIV-infected, virologically suppressed patients. BMC Infect Dis 2017;17:723.:723. 10.1186/s12879-017-2831-9 29145807 PMC5691866

[55] Kamal P, Sharma S. SUN-187 dolutegravir causing diabetes. Journal of the Endocrine Society 2019;3.(Supplement_1) 10.1210/js.2019-SUN-187

[56] Shah S, Hill A. Risks of metabolic syndrome and diabetes with integrase inhibitor-based therapy. Curr Opin Infect Dis 2021;34:16–24. 10.1097/QCO.0000000000000695 33278177

