# Supplementary material for: Association between integrase strand transfer inhibitor use with insulin resistance and incident diabetes mellitus in persons living with HIV: a systematic review and meta-analysis
Source: BMJ Open Diabetes Res Care. 2023 Feb 8;11(1):e003136. doi: 10.1136/bmjdrc-2022-003136 (PMC9923267; doi:10.1136/bmjdrc-2022-003136)
Supplement: Supplementary data [file bmjdrc-2022-003136supp001.pdf]

Association between integrase strand transfer inhibitor use with insulin resistance and incident diabetes mellitus in persons living with HIV: a systematic review and meta-analysis

Frank Mulindwa[1,2], Habiba Kamal[3,4], Barbara Castelnuovo[1], Dathan M. Byonanebye[5,6], Jean-Marc Schwarz[7], Robert Bollinger[8] and Nele Brusselaers[2,9,10]

Affiliations:

1- Makerere University Infectious Diseases Institute, Kampala, Uganda.
2- Global Health Institute, Antwerp University, Antwerp, Belgium
3- Department of Infectious Diseases, Karolinska University Hospital, Stockholm, Sweden.
4- Department of Medicine Huddinge, Karolinska Institute, Stockholm, Sweden.
5- Kirby Institute, University of New South Wales, Sydney, Australia
6- School of Public health, Makerere University, Kampala, Uganda.
7- University of California San Francisco, School of Medicine, San Francisco, USA
8- Johns Hopkins University, Baltimore, USA
9- Centre for Translational Microbiome Research, Department of Microbiology, Tumour and Cell Biology, Karolinska Institutet, Stockholm, Sweden.
10- Department of Head and Skin, Ghent University, Ghent, Belgium

Supplementary

SD: Link to the study dataset.

EMethods 1: Documentation of search strategies by Karolinska University Library search consultation group

Tables

- Table S1: Study characteristics of all the studies included in the systematic review and meta-analysis.
- Table S2: Outcome definitions.
- Table S3: Fields of collected data in excel sheet.
- Table S4: Quality assessment of the 12 randomized controlled trials included in the meta-analysis per Revised Cochrane risk-of-bias tool (RoB2)
- Table S5: Quality assessment of the 6 cohort studies included in the meta-analysis per Newcastle-Ottawa scale (NOS)*
- Table S6: Studies excluded upon full text review and reasons for exclusion.
- Table S7: Meta-regression* analysis of study and HIV-related variables on the pooled effect estimate

Figures

- Figure S1: Sub-analysis by non-INSTIs regimen in the control group (protease inhibitors and non-nucleotide reverse transcriptase inhibitors).
- Figure S2: Sub-analysis by follow-up duration
- Figure S3: Sub-analysis geographical origin of the study population.
- Figure S4: Sub-analysis by ART status at baseline.
- Figure S5: Sub-analysis by study design.

Mulindwa F, *et al. BMJ Open Diab Res Care* 2024; 11:e003136. doi: 10.1136/bmjdrc-2022-003136

- Figure S6: Assessment of publication bias by funnel plot asymmetry test for 13 studies meta-analysed
  for incident insulin resistance and/or diabetes in INSTIs compared to non-INSTIs.
- Figure S7. Mean changes in HOMA-IR from baseline in INSTIs group compared to overall non-INSTIs, NNRTIs and PIs groups.
- Figure S8: Bubble plots for univariable meta-regression on (A) Baseline viral load (B) Age (C) Baseline CD4 cell count.
- Figure S9: Bubble plot for univariable meta-regression on follow-up time.
- Figure S10: Influence analysis*by leave one-out-method for 13 studies pooled in the meta-analysis sorted by effect size.
- Figure S11: Baujat plot*showing each study contribution to the overall heterogeneity of the meta-analysis (studies n=13)
- S12; Link to dataset
- Continuation of manuscript references:

SD: https://osf.io/9eh74/?view_only=916713d9739340879e47249c8625b33d

EMethods 1: Documentation of search strategies by Karolinska University Library search consultation group

Date: 15th - June 2022

Topic/research question: Association between integrase strand transfer inhibitor use with insulin resistance and incident diabetes mellitus in persons living with HIV: a systematic review and meta-analysis

Name of researcher(s): Frank Mulindwa, Habiba Kamal & Nele Brusselaers.

Librarian(s): GunBrit Knutssön & Narcisa Hannerz

Databases:

1. Medline(OVID)
2. Embase.com
3. Web of Science(Clarivate)

Total number of hits:

- Before deduplication: 15,890
- After deduplication: 9,853

A) Medline

BMJ Publishing Group Limited (BMJ) disclaims all liability and responsibility arising from any reliance placed on this supplemental material which has been supplied by the author(s)

Interface: Ovid MEDLINE(R) and Epub Ahead of Print, In-Process & Other Non-Indexed Citations and Daily<br>Date of Search: 26th of January 2022<br>Number of hits: 3,930<br>Comment: In Ovid, two or more words are automatically searched as phrases, i.e. no quotation marks are needed	Field labels<br>• exp/ = exploded MeSH term<br>• / = non exploded MeSH term<br>• .ti,ab,kf. = title, abstract and author keywords<br>• adjx = within x words, regardless of order<br>• * = truncation of word for alternate endings

#	Searches	Results
1	exp Anti-Retroviral Agents/	84,542
2	Highly Active Antiretroviral Therapy/	11752
3	((agent* or drug* or inhibitor* or therapy) adj1 (anti-aids or anti-hiv or anti-retroviral or antiretroviral or hiv integrase)).ti,ab,kf.	59278
4	(bictegravir or cabotegravir or dolutegravir or elvitegravir or raltegravir or HAART).ti,ab,kf.	15983
5	or/1-4	115,852
6	Blood Glucose/	177,763
7	exp Diabetes Mellitus/	481,823
8	Glycated Hemoglobin A/	40338
9	Homeostasis/	68,901
10	exp Hyperglycemia/	39650
11	Hyperinsulinism/	8908
12	exp Insulins/	201259
13	exp Insulin Resistance/	94596
14	(diabetes or insulin-dependent or non-insulin dependent or prediabetic).ti,ab,kf.	618990
15	(blood glucose or fasting glucose or glucose intolerance or hemoglobin* or homeosta* or HOMA or hyperglyc?em* or insulin* or hyperinsulin*).ti,ab,kf.	821226
16	((complicat* or syndrome) adj1 (cardiometabolic or cardiovascular or dysmetabolic or metabolic or reaven)).ti,ab,kf.	76848
17	(DM1 or DM2 or IDDM or Hb A1 or HbA1 or Hb A1c or HbA1c or MODY or NIDDM or T1D or T2D).ti,ab,kf.	79840

18	or/6-17	1428922
19	5 and 18	4567
20	exp HIV Infections/	306184
21	(acquired immunodeficiency syndrome or AIDS or HIV).ti,ab,kf.	426783
22	or/20-21	468198
23	5 and 18 and 22	4271
24	limit 23 to yr="2000 -Current"	4034

B) Embase

Interface: embase.com<br><br>Date of Search: 15th of June 2022<br><br>Number of hits: 6,045<br><br>Comment: Emtree is the controlled vocabulary in Embase	Field labels<br><br>• /exp = exploded Emtree term<br>• /de = non exploded Emtree term<br>• ti,ab,kw = title, abstract and author keywords<br>• NEAR/x = within x words, regardless of order<br>• * = truncation of word for alternate endings

#1 'antiretrovirus agent'/exp/mj
#2 'highly active antiretroviral therapy'/mj
#3 ((agent* OR drug* OR inhibitor* OR therapy) NEAR/1 ('anti aids' OR 'anti hiv' OR 'anti retroviral' OR antiretroviral OR 'hiv integrase')):ti,ab,kw
#4 bictegravir:ti,ab,kw OR cabotegravir:ti,ab,kw OR dolutegravir:ti,ab,kw OR elvitegravir:ti,ab,kw OR raltegravir:ti,ab,kw OR haart:ti,ab,kw
#5 #1 OR #2 OR #3 OR #4#6 'glucose blood level'/mj
#7 'diabetes mellitus'/exp/mj
#8 'glycosylated hemoglobin'/exp/mj
#9 'homeostasis'/mj
#10 'hyperglycemia'/mj
#11 'glucose intolerance'/mj
#12 'hyperinsulinism'/mj
#13 'insulin derivative'/exp/mj
#14 'insulin resistance'/mj
#15 'metabolic syndrome x'/mj
#16 diabetes:ti,ab,kw OR 'insulin-dependent':ti,ab,kw OR 'non-insulin dependent':ti,ab,kw OR prediabetic:ti,ab,kw
#17 'blood glucose':ti,ab,kw OR 'fasting glucose':ti,ab,kw OR 'glucose intolerance':ti,ab,kw OR hemoglobin*:ti,ab,kw OR homeosta*:ti,ab,kw OR homa:ti,ab,kw OR hyperglyc$em*:ti,ab,kw OR insulin*:ti,ab,kw OR hyperinsulin*:ti,ab,kw
#18 ((complicat* OR syndrome) NEAR/1 (cardiometabolic OR cardiovascular OR dysmetabolic OR metabolic OR reaven)):ti,ab,kw
#19 'dm1':ti,ab,kw OR 'dm2':ti,ab,kw OR 'iddm':ti,ab,kw OR 'hb a1':ti,ab,kw OR 'hba1':ti,ab,kw OR 'hb a1c':ti,ab,kw OR 'hba1c':ti,ab,kw OR 'mody':ti,ab,kw OR 'niddm':ti,ab,kw OR 't1d':ti,ab,kw OR 't2d':ti,ab,kw
#20 #6 OR #7 OR #8 OR #9 OR #10 OR #11 OR #12 OR #13 OR #14 OR #15 OR #16 OR #17 OR #18 OR #19
#21 #5 AND #20

#22 'human immunodeficiency virus infection'/exp/mj
#23 'acquired immunodeficiency syndrome':ti,ab,kw OR aids:ti,ab,kw OR hiv:ti,ab,kw OR "human immunodeficiency virus $ infection':ti,ab,kw

Mulindwa F, *et al. BMJ Open Diab Res Care* 2024; 11:e003136. doi: 10.1136/bmjdrc-2022-003136

#24 #22 OR #23

#25 #21 AND #24 AND [2000-2022]/py

C) Web of Science Core Collection

Interface: Clarivate Analytics<br><br>Date of Search: 15th[th] of June 2022<br><br>Number of hits: 5,755	Field labels<br><br>• TS/Topic = title, abstract, author keywords and Keywords Plus<br>• NEAR/x = within x words, regardless of order<br>• * = truncation of word for alternate endings<br><br>Note: sometimes “quotation marks” are needed for single search terms to avoid automatic term mapping (lemmatization).

#1 ((agent* or drug* or inhibitor* or therapy) NEAR/1 (“anti-aids” or “anti-hiv” or anti-retroviral or antiretroviral or “hiv integrase”)) OR (bictegravir or cabotegravir or dolutegravir or elvitegravir or raltegravir or HAART)

#2 (diabetes or “insulin-dependent” or “non-insulin dependent” or prediabetic) OR

(“blood glucose” or “fasting glucose” or “glucose intolerance” or hemoglobin* or homeosta* or HOMA or hyperglyc$em* or insulin* or hyperinsulin*) OR ((complicat* or syndrome) NEAR/1 (cardiometabolic or cardiovascular or dysmetabolic or metabolic or reaven)) OR

(DM1 or DM2 or IDDM or “Hb A1” or HbA1 or “Hb A1c” or HbA1c or MODY or NIDDM or T1D or T2D)

#3 (“acquired immunodeficiency syndrome” or AIDS or HIV)

#4 #1 AND #2 AND #3 Refined by: PUBLICATION YEARS: 2000- 2022

Mulindwa F, *et al. BMJ Open Diab Res Care* 2024; 11:e003136. doi: 10.1136/bmjdrc-2022-003136

Table S1: Study characteristics of all the studies included in the systematic review and meta-analysis.

Study name	First author, Journal, year of publication	Study design, setting	Area of origin of study participants, (ART status at enrollment)	Outcome measure	Number on INSTIs*	Number on non-INSTIs regimen*	Duration of follow-up	Definition of diabetes mellitus	Definition of Insulin resistance	Reported potential conflict of interest
	Eron et al[54], The Lancet, 2010	RCT, multicenter	Africa, Asia, Europe, USA, Canada, Australia (ART experienced)	≥ grade 2 fasting hyperglycaemia⁎ (Not meta-analyzable)	350	352	24 weeks	≥ grade 2 fasting hyperglycaemia⁎	N/A	yes
	Saumoy et al[38], Atherosclerosis, 2012	RCT, multicenter	Spain (ART experienced)	Mean changes in HOMA-IR (Not meta-analyzable)	38	37	48 weeks	N/A	HOMA-IR	yes
**STARTMRK Trial**	Rockstroh et al[40], JAIDS, 2013	RCT, 67 sites in 5 continents	Europe/Australia, North America, Latin America, South East Asia (ART naïve)	RR	281	282	240 weeks	≥ grade 2 fasting hyperglycaemia⁎	N/A	yes
	Gupta et al[39], J Acquir Immune Defic Syndr, 2013	RCT, single center	USA (ART experienced)	Mean changes in HOMA-IR	15	15	48 weeks	N/A	HOMA-IR	yes
**ACTG Study A5257**	Lennox et al[43], Ann Intern Med., 2014	RCT, multicenter	USA, Puerto Rico (ART naïve)	RR	603	1208	96 weeks	≥ grade 2 fasting hyperglycaemia⁎	N/A	None for the current study
**FLAMINGO**	Clotet et al[41], Lancet, 2014	RCT, 64 research centers	France, Germany, Italy, Puerto Rico, Romania, Russia, Spain, Switzerland, and the USA (ART naïve)	RR	242	242	96 weeks	≥ grade 2 fasting hyperglycaemia⁎	N/A	yes
**SINGLE trial**	Walmsley et al[42], NEJM, 2015	RCT, multicenter	North America, Europe, Australia (ART naïve)	RR	414	419	48 weeks	≥ grade 2 fasting hyperglycaemia⁎	N/A	yes
	Fargo et al[44], Open Forum Infect Dis, 2016	RCT, multicenter	USA (ART naïve)	Mean changes in HOMA-IR	106	222	96 weeks	N/A	HOMA-IR	yes
	Spagnuolo et al[46], BMC Infectious Diseases, 2017	Cohort, single center	Italy (Mixed population)	RR	772	5423	462 weeks	Two consecutive FPG ≥126 mg/dl OR a 2-h OGTT plasma glucose	N/A	None for the current study

								level ≥200 mg/dL OR two consecutive fasting HBA1C levels of ≥48 mmol/mol, or a prescription for any antidiabetic medication		
**ANRS 12313 trial**	Delaporte et al[48], (NAMSAL study group), NEJM, 2019	RCT, multicenter	Cameroon (ART naïve)	RR	310	303	48 weeks	≥ grade 2 fasting hyperglycaemia⁎	N/A	None for the current study
	Gianotti et al[45], J Med Vir., 2019	Cohort, single center	Italy (ART naïve)	Mean changes in HOMA-IR	218	190 NNRTI, 210 PI/R	48 weeks	N/A	HOMA-IR	yes
	Ursenbach et al[28], Journal of Antimicrobial Chemotherapy, 2020	Cohort, multicenter in France and overseas	France (ART naïve)	RR	3403	16059	Variable	Documentation of diabetes in medical record, HbA1c > 7.5%, being on DM treatment	N/A	None for the current study
	Rebeiro et al[30], Clinical Infectious Diseases, 2020	Cohort, multicenter in North America	USA, Canada (ART naïve)	RR	5183	17701	Variable	HbA1c ≥6.5%, initiation of diabetes-specific medication, or new DM diagnosis	N/A	yes
**ADVANCE trial**	Venter et al[50], Lancet HIV, 2020	RCT, 11 public health clinics	South Africa (ART naïve)	RR	690	347	96 weeks	Not stated	N/A	yes
**INSPIRING study**	Dooley et al[47], Clinical Infectious Diseases, 2020	RCT, multicenter	Argentina, Brazil, Mexico, Peru, Russia, South Africa, and Thailand (ART naïve)	RR	69	44	52 weeks	≥ grade 2 fasting hyperglycaemia⁎	N/A	yes
	Hsu et al[49], AIDS, 2021	Cohort, 84 multicenter	USA (ART naïve and experienced)	RR	15122	2076	Variable	Recorded diagnosis of T2DM, anti-diabetic medication prescription, lab tests indicative of DM.	N/A	yes
**TANGO study**	Wyk et al[52], JAIDS, 2021	RCT, 134 multicenter in 10 countries	USA, Australia, Europe (ART experienced)	RR	303	290	48 weeks	NA	N/A	yes

	Ibrahim et al[51], HIV med., 2021	RCT, 9 HIV clinics in UK	UK (ART experienced)	Mean changes in HOMA-IR (Not meta-analysable)	34	19	48 weeks	N/A	HOMA-IR	yes
	Asundi et al[53], AIDS Res Hum Retroviruses, 2022	Cohort, Single center	USA (ART naïve)	Adjusted RR	136	1099	18 months	a new prescription for antihyperglycemic medication	N/A	None for the current study

Abbreviations: RCT=randomized controlled trial. ART=antiretroviral therapy. INSTIS=integrase strand transfer inhibitors. HOMA-IR: Homeostatic model of Insulin Resistance. RR= relative risk. N/A= not available. DM=diabetes mellitus. *The numbers represent patients without diabetes mellitus at baseline enrolled in the metabolic analyses in each study. NB Eron et al (54) not included in the metanalyses

Table S2. Outcome definitions

Study outcome	Acceptable outcome measures in individual studies included in the meta-analysis
**Diabetes mellitus**	1- ADA criteria(1): HbA1C ≥6.5% or FPG ≥126 mg/dL (7.0 mmol/L) or 2-h PG ≥200 mg/dL (11.1 mmol/L) during an OGTT<br>2- WHO criteria(2):<br>- fasting plasma glucose values of ≥ 7.0 mmol/L (126 mg/dl) OR<br>- 2-h post-load plasma glucose ≥ 11.1 mmol/L (200 mg/dl) OR<br>- HbA1c ≥ 6.5% (48 mmol/mol) OR<br>- random blood glucose ≥ 11.1 mmol/L (200 mg/ dl) in the presence of signs and symptoms Need for diabetes medication
**Metabolic syndrome**	1-NCEP ATP III criteria(3): The presence of three or more of the following risk determinants:<br>- increased waist circumference (>102 cm [>40 in] for men, >88 cm [>35 in] for women);<br>- elevated triglycerides (≥150 mg/dl);<br>- low HDL cholesterol (<40 mg/dl in men, <50 mg/dl in women);<br>- hypertension (≥130/≥85 mmHg); and<br>- *5*) impaired fasting glucose (≥110 mg/dl)<br><br>2-WHO criteria(4): Glucose intolerance, DM2 or insulin-resistance in addition to at least two of the following:<br>- BMI > 30 and HWR > 0.9 (M) and > 0.85 (F)<br>- Serum TG ≥ 150mg/dl<br>- Serum HDL < 35mg/dl (M), <39mg/dl (F)<br>- Blood pressure ≥ 140/90 or on hypertension treatment<br>- Other risk factors: microalbuminuria ≥20mcg/min<br><br>3-IDF(5): DM/ Glucose intolerance and two or more criteria<br>- Fasting glucose of 100-125mg/dl Or DM 2<br>- WC ≥ 94cm (M), 80cm (F)<br>- TG ≥150mg/dl<br>- HDL <40mg/dl or <50mg/dl<br>- On treatment for SAH/ BP ≥130/85mmHG<br><br>4-European Group for Study of Insulin Resistance definition(6): Elevated plasma insulin (>75th percentile) plus two other factors from among the following:<br>- Abdominal obesity: waist circumference (WC) ≥94 cm in men and ≥80 cm in women<br>- Hypertension: ≥140/90 mm of Hg or on antihypertensive treatment<br>- Elevated triglycerides (≥150 mg/dl) and/or reduced HDL-C (<39 mg/dl for both men and women)<br>- Elevated plasma glucose: impaired fasting glucose (IFG) or IGT, but no diabetes
**Insulin resistance**	Homeostatic model for Insulin resistance (HOMA-IR)(7)

Abbreviations: ADA= American Diabetes Association. HbA1C= Glycated Hemoglobin. PG= Plasma Glucose, OGTT= Oral Glucose Tolerance test. WHO= World Health Organization. NCEP ATP III= National Cholesterol Education Program Adult Treatment Panel III. HDL= High Density Lipoproteins. DM2= Diabetes Mellitus type II. BMI= Body Mass Index. TG= triglycerides. IDF=International Diabetes Federation. WC= Waist Circumference. SAH= Systemic Arterial Hypertension. BP= Blood Pressure. IFG= Impaired Fasting Glucose. IGT= Impaired Glucose Tolerance. HOMA-IR=Homeostatic Model for Insulin Resistance.

Table S3: Fields of data collection in excel sheet

Study title	Study ID	Author	Year of publication	Journal	Country	study design	geographical region of the cohort	continent	Setting (nationwide, register based, hospital based)
							Baseline		
Center (primary/ tertiary)	Study period	Recruitment duration	Inclusion criteria	Exclusion criteria	Overall cohort number	Duration of follow up	Number of patients per arm	Number ART naïve pts per arm	Previous exposure to ART duration per arm
Baseline									
number of patients per arm	Number ART naïve pts per arm	Previous exposure to ART duration per arm	ethnicity	at baselinen, % male %	Female n, %	composite age (mean/ median)	Age (mean, SD) or median male	Age (mean, SD) or median female	BMI
Baseline									
BMI women	BMI men	smoking	waist circumference	Presence of lipodystrophy, n, %	Presence of diabetes mellitus or treatment for DM	criteria of DM diagnosis	presence of MS (yes/no)	criteria of diagnsois of MS	Fasting blood glucose (mg/dl)
Baseline									
HbA1C	Insulin resistance	CD4 count (median/mean, SD)	HIV viral load	comorbidities viral hepatitis, n, %	comorbid ities tuberculo sis, n, %	AIDS			
Outcome									
outcome definition in the study	diagnostic methods of outcome	outcome number, % who developped diabetes per arm	Change in IR	number who developped metabolic syndrome, n%	change in blood sugar level, mean (SD)	change in Hb A1C level, mean (SD)	crude risk/ ?outcome	adjsuted risk /?outcome	Predictors adjusted for in the study

Abbreviations: ART=antiretroviral therapy. BMI=body mass index. MS= metabolic syndrome. HbA1C=glycated hemoglobin subtype 1C. IR=incidence rate.

Mulindwa F, *et al. BMJ Open Diab Res Care* 2024; 11:e003136. doi: 10.1136/bmjdrc-2022-003136

Table S4: Quality assessment of the 12 randomized controlled trials included in the meta-analysis

Study	Random sequence generation (selection bias)	Allocation concealment (selection bias)	Selective reporting (reporting bias)	Other sources of bias	Blinding (participants and personnel) - (performance bias)	Blinding (outcome assessment)- (detection bias)	Incomplete outcome data (attrition bias)
Saumoy et al, 2012	🔵	🔵	🔵	🔵	🔴	🔴	⚪
Rockstroh et al, 2013	🔵	🔵	🔵	🔵	🔵	⚪	🔵
Gupta et al, 2013	🔵	🔵	🔵	🔵	🔴	🔵	🔵
Clotet et al, 2014	🔵	🔵	🔵	🔵	🔴	🔴	🔴
Lennox et al, 2015	🔵	🔵	🔵	🔵	🔴	⚪	🔴
Walmsley et al, 2015	🔵	🔵	🔵	🔵	🔵	⚪	🔵
Fargo et al, 2016	🔵	🔵	🔵	🔵	🔴	🔴	⚪
Delaporte et al, 2019	🔵	🔵	🔵	🔵	🔴	⚪	🔵
Dooley et al, 2019	🔵	🔵	🔵	🔵	🔴	🔴	🔴
Venter et al, 2020	🔵	🔵	🔵	🔵	🔴	⚪	🔵
Wyk et al, 2021	🔵	🔵	🔵	🔵	🔴	⚪	🔵
Ibrahim et al, 2021	🔵	🔵	🔵	🔵	🔴	⚪	🔵

🔵 = low risk, 🔴 = high risk, ⚪ = unclear

*RoB 2: A revised Cochrane risk-of-bias tool for randomized trials | Cochrane Bias. https://methods.cochrane.org/bias/resources/rob-2-revised-cochrane-risk-bias-tool-randomized-trials

per Revised Cochrane risk-of-bias tool (RoB2) *

Table S5: Quality assessment of the 6 cohort studies included in the meta-analysis per Newcastle-Ottawa scale (NOS)**

	Selection				Comparability	Outcome			
**Study**	**1) Representativeness of the exposed cohort**	**2) Selection of the non-exposed cohort**	**3) Ascertainment of exposure**	**4) Demonstration that outcome of interest was not present at start of study**	**1) Comparability of cohorts based on the design or analysis controlled for confounders**	**1) Assessment of outcome**	**2) Was follow-up long enough for outcomes to occur**	**3) Adequacy of follow-up of cohorts**	**Overall score**
Spagnuolo et al, 2017	*	*	*	*	*	*	*	*	8
Gianotti et al, 2017	*	*	*	*	*	*	*	*	8
Ursenbach et al, 2020	*	*	*	*	*	*	*	*	8
Hsu et al, 2020	*	*	*	*	**	*	*	*	9
Rebeiro et al, 2020	*	*	*	*	**	*	*	*	9
Asundi et al, 2022	*	*	*	*	*	*			6

** **Newcastle-Ottawa scale (NOS)** Ottawa Hospital Research Institute. http://www.ohri.ca/programs/clinical_epidemiology/oxford.asp
Score ≥7 represents low risk of bias

Mulindwa F, *et al. BMJ Open Diab Res Care* 2024; 11:e003136. doi: 10.1136/bmjdrc-2022-003136

Table S6: Studies excluded upon full text review and reasons for exclusion

Study title	Year	Journal	Author	Reason for exclusion
1. Simplification from protease inhibitors to once- or twice-daily raltegravir: the ODIS trial	2010	HIV clinical trials	Vispo et al	Single arm study. Additionally, no outcome
2. Substitution of raltegravir for ritonavir-boosted protease inhibitors in HIV-infected patients: the SPIRAL study.	2010	AIDS	Martinez et al	No outcome of interest
3. Simplification from protease inhibitors to once- or twice-daily raltegravir: the ODIS trial	2010	HIV Clin Trials	Vispo et al	No outcome of interest
4. Switch to a raltegravir-based regimen versus continuation of a lopinavir-ritonavir-based regimen in stable HIV-infected patients with suppressed viraemia (SWITCHMRK 1 and 2):	2010	The Lancet	Eron et al	Outcome not studied
5. Durability of a novel salvage therapy in R5 HIV-infected patients: Maraviroc, raltegravir, etravirine	2011	J Acquir Immune Defic Syndr	Nozza et al	No outcome of interest
6. Switching antiretroviral therapy to minimize metabolic complications	2011	HIV Therapy	Lake et al	Narrative systematic review
7. Raltegravir as replacement for PI- or NNRTIbased ART in HIV-infected women with lipohypertrophy: The Women, Integrase, and Fat Accumulation Trial	2011	Antiviral Therapy	Lake et al	No outcome of interest
8. Long-term glucose tolerance in highly experienced HIV-infected patients receiving nucleoside analogue-sparing regimens	2012	AIDS (London, England)	Bigoloni et al	INSTI vs INSTI
9. Elvitegravir/cobicistat /emtricitabine /tenofovir DF (Quad) has noninferior efficacy and favorable safety compared to efavirenz/emtricitabine/tenofovir df in treatment naive HIV-1	2012	Canadian journal of infectious diseases and medical	Sax et al	No outcome of interest
10. A Randomized Trial of Raltegravir Replacement for Protease Inhibitor or Non-Nucleoside Reverse Transcriptase Inhibitor in HIV-Infected Women with Lipohypertrophy	2012	Aids patients care and STDs	Lake et al	No outcome of interest
11. Changes in cardiovascular biomarkers in HIV-infected patients switching from ritonavir-boosted protease inhibitors to raltegravir	2012	AIDS	Martinez et al	No outcome of interest
12. Co-formulated elvitegravir, cobicistat, emtricitabine, and tenofovir disoproxil fumarate versus ritonavir-boosted atazanavir plus co-formulated emtricitabine and tenofovir disoproxil	2012	Lancet	DeJesus et al	Diabetes patients were not excluded at baseline
13. Cardiovascular risk in human immunodeficiency virus-infected patients in Spain. CoRIS cohort	2012	Enferm Infecc Microbiol Clin	Masia et al	No outcome of interest
14. A randomised trial of Raltegravir Replacement for Protease Inhibitor or Non-Nucleoside Reverse Transcriptase Inhibitor in HIV Infected Women with Lipohypertrophy	2012	AIDS Patient Care and STDs	Lake et al	Outcome not studied
15. Once-daily dolutegravir versus twice-daily raltegravir in antiretroviral-naive adults with HIV-1 infection (SPRING-2 study): 96 week results from a randomised, double-blind, non-	2013	lancet infectious diseases	Raffi et al	Both treatment arms contained INSTIs
16. HIV lipodystrophy in participants randomised to lopinavir/ritonavir (LPV/r) +2-3 nucleoside/nucleotide reverse transcriptase inhibitors (N(t)RTI) or LPV/r + raltegravir as	2013	PloS one	Martin et al	Protease inhibitors coupled with INSTI in
17. Effects of switching from stavudine to raltegravir on subcutaneous adipose tissue in HIV-infected patients with HIV/HAART-associated lipodystrophy syndrome (HALS). A clinical	2014	PloS one	Domingo et al	Single arm study
18. Dolutegravir: clinical and laboratory safety in integrase inhibitor-naive patients	2014	HIV Clin Trials	Curtis et al	Narrative review

19. A randomized, double-blind comparison of single-tablet regimen elvitegravir/cobicistat/emtricitabine/tenofovir DF vs ritonavir-boosted atazanavir plus	2014	J Acquir Immune Defic Syndr	Clumek et al	No outcome of interest
20. Simplification to coformulated elvitegravir, cobicistat, emtricitabine, and tenofovir versus continuation of ritonavir-boosted protease inhibitor with emtricitabine and teno fovir in adults	2014	Lancet Infect Dis	Arribas et al	No outcome of interest
21. A randomized, double-blind comparison of single-tablet regimen elvitegravir/cobicistat/emtricitabine/tenofovir DF versus single-tablet regimen	2014	J Acquir Immune Defic Syndr	Wohl et al	No outcome of interest
22. The impact of switching from protease-inhibitor to integrase-inhibitor therapy on biomarkers of metabolic and age-associated diseases: A longitudinal matched-cohort study	2014	Antiviral Therapy	Malagoli et al	No outcome of interest
23. Telmisartan to reduce cardiovascular risk in older HIV-infected adults: a pilot study	2015	HIV clinical trials	Lake et al	No outcome of interest
24. A prospective, randomized clinical trial of antiretroviral therapies on carotid wall thickness	2015	AIDS	Stein et al	No outcome of interest
25. Comparative changes of lipid levels in treatment-naive, HIV-1-infected adults treated with dolutegravir vs. efavirenz, raltegravir, and ritonavir-boosted darunavir-based regimens over	2015	Clinical drug investigation	Quercia et al	No outcome of interest
26. Comparison of the metabolic effects of ritonavir-boosted darunavir or atazanavir versus raltegravir, and the impact of ritonavir plasma exposure: ACTG 5257	2015	Clinical infectious diseases	Ofotokun et al	No outcome of interest
27. Post-prandial lipid effects of raltegravir versus darunavir/ritonavir in HIV-1-infected adults commencing combination ART	2015	J Antimicrob Chemother	Lee et al	No outcome of interest
28. Effects of raltegravir combined with tenofovir/emtricitabine on body shape, bone density, and lipids in African-Americans initiating HIV therapy	2015	HIV clinical trials	Young et al	Single arm study
29. Human immunodeficiency virus (HIV) modulates the associations between insulin resistance and cognition in the current combination antiretroviral therapy (cART) era: a	2015	Journal of neurovirology	Valcour et al	No outcome of interest
30. Rosuvastatin vs. protease inhibitor switching for hypercholesterolaemia: a randomized trial	2016	HIV medicine	Lee et al	No outcome of interest
31. Efficacy of dolutegravir/abacavir/lamivudine (DTG/ABC/3TC) fixed-dose combination (FDC) compared with ritonavir-boosted atazanavir (ATV/R) plus tenofovir disoproxil	2016	Open Forum Infect Dis	Hagins	No outcome of interest
32. Integrase inhibitor versus protease inhibitor-based regimen for HIV-1 infected women (WAVES): a randomised, controlled, double-blind, phase 3 study.	2016	Lancet HIV	Squires et al	No outcome of interest
33. Efficacy and Safety of Elvitegravir/Cobicistat/Emtricitabine/ Tenofovir Disoproxil Fumarate in Asian Subjects with Human Immunodeficiency Virus 1 Infection: A SubAnalysis of Phase 3	2016	Infect Chemother	Choi et al	Sub-analysis of already included studies
34. Switching to Tenofovir Alafenamide, Coformulated With Elvitegravir, Cobicistat, and Emtricitabine, in HIV-Infected Patients With Renal Impairment: 48-Week Results From a	2016	Journal of acquired immune deficiency syndromes	Pozniak et al	Single arm study
35. Neither boosted elvitegravir nor darunavir with emtricitabine/tenofovir disoproxil fumarate increase insulin resistance in healthy volunteers: results from the STRIBILD-IR study	2016	Antiviral therapy	Spinner et al	HIV negative study participants
36. Switch to dolutegravir in HIV patients responding to a firstline antiretroviral treatment: 48 weeks results	2016	J Int AIDS Soc	Tau et al	No outcome of interest
37. Changes in liver steatosis after switching efavirenz to raltegravir: The steral study	2017	Clin Infect Dis	Macias et al	No outcome of interest
38. Weight Gain in Persons with HIV Switched from Efavirenz-Based to Integrase Strand Transfer Inhibitor-Based Regimens	2017	JAIDS	Norwood et al	No outcome of interest

Mulindwa F, *et al. BMJ Open Diab Res Care* 2024; 11:e003136. doi: 10.1136/bmjdrc-2022-003136

39. Phase 3 randomized, controlled trial of switching to fixed-dose bictegravir/emtricitabine/tenofovir alafenamide (B/F/TAF) from boosted protease inhibitor-	2017	Open Forum Infec Dis	Daar et al	No outcome of interest
40. Switching from a ritonavir-boosted protease inhibitor to a dolutegravir-based regimen for maintenance of HIV viral suppression in patients with high cardiovascular risk.	2017	AIDS	Gatell et al	Comparator groups had diabetic patients at
41. Switching to the single-tablet regimen of elvitegravir, cobicistat, emtricitabine, and tenofovir DF from non-nucleoside reverse transcriptase inhibitor plus co-formulated emtricitabine and	2017	HIV Clin Trials	Pozniac et al	No outcome of interest
42. Fixed-dose combination dolutegravir, abacavir, and lamivudine versus ritonavir-boosted atazanavir plus tenofovir disoproxil fumarate and emtricitabine in previously untreated	2017	Lancet HIV	Orrell et al	No outcome of interest
43. Impact on lipid abnormalities of switching from a ritonavir-boosted protease inhibitor to a raltegravir-based cART regimen	2017	AIDS	Gatell et al	No outcome of interest
44. Body composition and metabolic outcomes after 96 weeks of treatment with ritonavir-boosted lopinavir plus either nucleoside or nucleotide reverse transcriptase inhibitors or	2017	Lancet HIV	Boyd et al	Both comparator groups had patients on INSTIs.
45. Adiponectin and the steatosis marker Chi3L1 decrease following switch to raltegravir compared to continued PI/NNRTI-based antiretroviral therapy	2018	PloS one	Offor et al	No outcome of interest
46. Changes in Waist Circumference in HIV-Infected Individuals Initiating a Raltegravir or Protease Inhibitor Regimen: Effects of Sex and Race	2018	Open forum infectious diseases	Bhagwat et al	No outcome of interest
47. Effects of antiretroviral combination therapies F/TAF, E/C/F/TAF and R/F/TAF on insulin resistance in healthy volunteers: the TAF-IR Study	2018	Antiviral therapy	Spinner et al	Non-HIV population
48. Gestational diabetes in women on dolutegravir- or efavirenz-based ART in Botswana	2018	Topics in Antiviral Medicine	Mmasa et al	Pregnant population
49. Durability and tolerability of first-line combination including two NRTI and RAL or ATV/r or DRV/r in patients enrolled in the ICONA Foundation cohort. HIV Drug Therapy, Glasgow	2018	HIV Clinical Trials	Monforte et al	No outcome of interest
50. Evaluation of the efficacy and safety of integrase inhibitor in the treatment of acute HIV infection.	2018	ChiCtr	Kang et al	Protocol publication. No results posted yet
51. Dolutegravir + lamivudine dual therapy in patients with suppressed HIV-RNA: Long term virological and immunological results of a multicentre cohort	2018	Journal of the International AIDS Society	Maggiolo et al	Single arm, no comparator group
52. Lower pretreatment gut integrity associated with fat gains on antiretrovirals	2018	Topics in Antiviral Medicine	Kamari et al	No outcome of interest
53. Obesity following ART initiation is common and influenced by both traditional and HIV-/ART-specific risk factors	2018	The Journal of antimicrobial chemotherapy	Bakal et al	No outcome of interest
54. Diagnostic Accuracy of Noninvasive Markers of Steatosis, NASH, and Liver Fibrosis in HIV-Monoinfected Individuals at Risk of Nonalcoholic Fatty Liver Disease (NAFLD): Results from	2019	Journal of acquired immune deficiency syndromes	Lemoine et al	No outcome of interest
55. Evaluation of Safety and Effectiveness of Elvitegravir/Cobicistat/Emtricitabine/Tenofovir Alafenamide Switch Followed by Ledipasvir/Sofosbuvir HCV Therapy in HIV-HCV	2019	Open forum infectious diseases	Doyle et al	No outcome of interest
56. Improvement in liver steatosis after the switch from a ritonavir-boosted protease inhibitor to raltegravir in HIV-infected patients with non-alcoholic fatty liver disease	2019	Infectious diseases (London, England)	Calza et al	No outcome of interest
57. Metabolic, mitochondrial, renal and hepatic safety of enfuvirtide and raltegravir antiretroviral administration: Randomized crossover clinical trial in healthy volunteers	2019	PloS one	Barosso et al	HIV negative population
58. Reduced soluble CD14 levels after switching from a dual regimen with lamivudine plus boosted protease inhibitors to lamivudine plus dolutegravir in virologically suppressed HIV-	2019	HIV Res Clin Pract	Lombardi et al	No outcome of interest

59. Body composition and adipokines changes after initial treatment with darunavir-ritonavir plus either raltegravir or tenofovir disoproxil fumarate-emtricitabine: A substudy of the NEAT001/ANRS143 randomised trial	2019	PloS one	Bernadino et al	One arm had a combination of a protease inhibitor and [illegible]
60. Changes in Lipid Indices in HIV+ Cases on HAART	2019	BioMed research international	Ji et al	No outcome of interest
61. Improvement in insulin sensitivity and serum leptin concentration after the switch from a ritonavir-boosted PI to raltegravir or dolutegravir in non-diabetic HIV-infected patients	2019	The Journal of antimicrobial chemotherapy	Calza et al	Both study arms had INSTIs
62. Incidence of select chronic comorbidities among a population-based cohort of HIV-positive individuals receiving highly active antiretroviral therapy	2019	Current medical research and opinion	Gali et al	No outcome of interest
63. Lipid profile improvement in virologically suppressed HIV-1-infected patients switched to dolutegravir/abacavir/lamivudine: data from the SCOLTA project	2019	Infection and drug resistance	Bagella et al	No outcome of interest
64. Short-term increase in Body Mass Index and systolic blood pressure elevation in treatment naÃ¯ ve persons starting INSTI based antiretroviral therapy	2019	HIV Medicine	Galdamez et al	Single arm study (no comparator group)
65. Dolutegravir versus ritonavir-boosted lopinavir both with dual nucleoside reverse transcriptase inhibitor therapy in adults with HIV-1 infection in whom first-line therapy has	2019	Lancet Infectious Diseases	Aboud et al	Diabetic patients were not excluded at baseline
66. Efficacy and safety of switching to coformulated elvitegravir, cobicistat, emtricitabine, and tenofovir alafenamide (E/C/F/TAF) in virologically suppressed women	2019	J Acquir Immune Defic Syndr.	Hodder et al	No outcome of interest
67. Effects of integrase strand-transfer inhibitor use on lipids, glycemic control, and insulin resistance in the women's interagency HIV study (WIHS)	2019	Open Forum Infect Dis	Aldredge et al	No outcome of interest
68. Exploring the Prevalence and Characteristics of Weight Gain and other Metabolic Changes in Patients with HIV Infection Switching to Integrase Inhibitor Containing ART	2019	Open Forum Infect Dis.	Zimmerman et al	No outcome of interest
69. Switching from boosted protease inhibitors (PI/r) to dolutegravir (DTG) in virologically suppressed HIV-infected patients with high cardiovascular risk: 48-week effects on	2020	J Antimicrob Chemother	Gonzalez et al	No outcome of interest
70. Comorbidities, antiretroviral therapy switches, and drug side-effects among HIV-infected patients	2020	Klimik Dergisi	Evlice et al	No outcome of interest
71. Dolutegravir-associated hyperglycaemia in patients with HIV	2020	The lancet. HIV	Mohammed et al	Outcome definition was symptomatic
72. Early scale-up of antiretroviral therapy at diagnosis for reducing economic burden of cardiometabolic disease in HIV-infected population	2020	AIDS	Yang et al	No outcome of interest
73. Factors Associated With Weight Gain in People Treated With Dolutegravir	2020	Open forum infectious diseases	Taramasso et al	No outcome of interest
74. Greater Weight Gain in Treatment-naive Persons Starting Dolutegravir-based Antiretroviral Therapy	2020	Clinical infectious diseases	Bourgi et al	No outcome of interest
75. Metabolic Changes Associated With the Use of Integrase Strand Transfer Inhibitors Among Virally Controlled Women	2020	Journal of acquired immune deficiency syndromes	Summers et al	Both comparator groups had diabetic patients at baseline
76. Real-World Assessment of Weight Change in People with HIV-1 After Initiating Integrase Strand Transfer Inhibitors or Protease Inhibitors	2020	Journal of health economics and outcomes research	Chen et al	No outcome of interest
77. Risk Factors for Weight Gain Following Switch to Integrase Inhibitor-Based Antiretroviral Therapy	2020	Clinical infectious diseases	Lake et al	No outcome of interest

78. The association between HIV tri-therapy with the development of Type-2 Diabetes Mellitus in a rural South African District: A case-control study	2020	PLOS one	Bam et al	Case control design was among exclusion criteria
79. Durable Suppression and Low Rate of Virologic Failure 3 Years After Switch to Dolutegravir + Rilpivirine 2-Drug Regimen: 148-Week Results From the SWORD-1 and SWORD-2	2020	J Acquir Immune Defic Syndr	Jean van Wyk et al	One arm had an INSTI and NNRTI combined
80. Fat gain differs by sex and hormonal status in persons living with suppressed HIV switched to raltegravir/etravirine	2020	AIDS	Assoumou et al	INSTIs and NNRTIs in the same arm
81. Plasma lipidome abnormalities in people with HIV initiating antiretroviral therapy	2020	Translational Medicine Communications	Bowman et al	No outcome of interest
82. Weight gain and dyslipidaemia among virally suppressed HIV-positive patients switching to co-formulated elvitegravir/cobicistat/emtricitabine/tenofovir alafenamide	2020	International journal of infectious diseases	Kuo et al	No outcome of interest
83. Weight Gain Associated With Integrase Stand Transfer Inhibitor Use in Women	2020	Clinical Infectious Diseases	Kerchberger et al	No outcome of interest
84. Weight gain during pregnancy in women with HIV receiving different antiretroviral regimens	2020	Antiviral therapy	Floridia et al	Pregnant study participants
85. Weight gain following antiretroviral therapy (ART) initiation in ART-naive participants in the current treatment era	2020	Pharmacoepidemiology and Drug Safety	Ruderman et al	No outcome of interest
86. Weight gain in persons living with HIV (PLWH) treated with bictegravir compared to other integrase strand transfer inhibitors	2020	Open Forum Infectious Diseases	Fang et al	INSTI vs INSTI study
87. Excess burden of age-associated comorbidities among people living with HIV in British Columbia, Canada: a population-based cohort study	2021	BMJ open	Nanditha et al	Compared HIV patients to HIV negative patients.
88. The burden of non-communicable diseases and mortality in people living with HIV (PLHIV) in the pre-, early- and late-HAART era	2021	HIV medicine	Jespersen et al	Comparative group is a non-HIV population
89. Gestational diabetes in women living with HIV in Botswana: lower rates with dolutegravir- than with efavirenz-based antiretroviral therapy	2021	HIV medicine	Mmasa et al	Pregnant population
90. Weight changes after antiretroviral therapy initiation in CoRIS (Spain): a prospective multicentre cohort study	2021	JIAS	Martinez et al	No outcome of interest
91. Antiretroviral Therapy Initiation Is Associated With Decreased Visceral and Subcutaneous Adipose Tissue Density in People Living With Human Immunodeficiency Virus	2021	Clinical infectious diseases	Debroy et al	No outcome of interest
92. Brief Report: Weight Gain Following ART Initiation in ART-Naive People Living With HIV in the Current Treatment Era	2021	Journal of acquired immune deficiency syndromes	Ruderman et al	No outcome of interest
93. Effect of menopause on weight gain, insulin and waist circumference in women with HIV who switch antiretroviral therapy to abacavir/lamivudine/dolutegravir	2021	AIDS	Hamzah et al	No outcome of interest
94. Implications of weight gain with newer antiretrovirals: 10-year predictions of cardiovascular disease and diabetes	2021	AIDS	McCann et al	No outcome of interest
95. Changes in renal and metabolic indices after switching from tenofovir disoproxil fumarate- to tenofovir alafenamide-containing ART among individuals with HIV in Canada: A	2021	International journal of STD & AIDS	Shokoohi et al	No outcome of interest
96. Antiretroviral Therapy Initiation Is Associated With Decreased Visceral and Subcutaneous Adipose Tissue Density in People Living With Human Immunodeficiency Virus	2021	Clin Infect Dis	Debroy et al	No outcome of interest
97. Insulin resistance in people living with HIV is associated with exposure to thymidine analogues and/or didanosine and prior immunodeficiency	2022	BMC Infectious Diseases	Høgh et al	No outcome of interest

98. Two decade trends in cardiovascular disease risk factor and outcome burden among veterans with HIV	2022	J. Am. Coll. Cardiol.	Haji et al	No outcome of interest
99. Weight changes, metabolic syndrome and all-cause mortality among Asian adults living with HIV	2022	HIV Med.	Han et al	No outcome of interest
100. Longitudinal analysis of new-onset non-AIDS-defining diseases among people living with HIV: A real-world observational study	2022	HIV Med	Duan et al	No outcome of interest
101. Metabolic complications of highly active antiretroviral therapy in adult HIV-infected patients with heart failure: A 7-year prospective cohort study	2022	Metab. Clin. Exp.	Ma et al	Conference abstract
102. Abacavir antiretroviral therapy and indices of subclinical vascular disease in persons with HIV	2022	PLoS ONE	Martinez et al	No INSTI group
103. Adipokines, Weight Gain and Metabolic and Inflammatory Markers After Antiretroviral Therapy Initiation: AIDS Clinical Trials Group (ACTG) A5260s	2022	Clin. Infect. Dis.	Koethe et al	No outcome of interest
104. The risk of hyperglycaemia associated with the use of dolutegravir among adults living with HIV in Kampala, Uganda: a case-control study	2022	Lancet Global Health	Namara et al	Design excluded
105. Estimating atherosclerotic risk in south african youth with perinatally acquired HIV	2022	Top. Antiviral Med.	Mahtab et al	Could not retrieve record
106. Atrial fibrillation risk factors among patients in hiv care in the United States	2022	Top. Antiviral Med.	Nance et al	No outcome of interest
107. Trends in myocardial infarction risk by hiv status in 2 US healthcare systems	2022	Top. Antiviral Med.	Silverberg et al	Poster
108. InSTI-related body composition differences in chronically infected MLWH	2022	Top. Antiviral Med.	Wisch et al	Poster
109. Metabolic Profile of People Living with HIV in a Treatment Hub in Manila, Philippines: A Pre- and Post-Antiretroviral Analysis	2022	J. ASEAN Fed. Endocr. Soc.	Francisco et al	No INSTI arm
110. Metabolic comorbidities and systemic arterial hypertension: the challenge faced by HIV patients on long-term use of antiretroviral therapy	2022	Hosp Pract (1995)	Mendicino et al	Cross sectional study
111. Evaluation of cardiotoxicity and other adverse effects associated with concomitant administration of artemether/lumefantrine and atazanavir/ritonavir-based antiretroviral regimen in patients living with HIV	2022	Saudi Pharm. J.	Usman et al	No outcome of interest
112. Real-World Characterization of the Portuguese Population Living with HIV who Initiated Raltegravir Based-Regimens: The REALITY Study	2022	Acta Med. Port.	SerrÃ£o et al	Single arm, no comparator group
113. Real life use of dolutegravir doravirine dual regimen in experienced elderly PLWH with multiple comorbidities and on polypharmacy A retrospective analysis	2021	Medicine	Mazzitelli et al	Single arm
114. Factors associated with cardiometabolic parameters at 3 years in the TANGO Study, comparing a switch to dolutegravir/ lamivudine versus maintenance of tenofovir [illegible]	2021	Antiviral Ther.	Batterhan et al	Poster
115. Integrase strand transfer inhibitors are associated with higher blood pressure and renin-angiotensin-aldosterone system activity	2021	Antiviral Ther.	Siddiqui et al	No outcome of interest
116. Increase in pro-atherogenic apolipoprotein B in people living with HIV (PLWH) following switch from tenofovir disoproxil fumarate to tenofovir alafenamide	2021	Antiviral Ther.	Savinelli et al	No outcome of interest
117. Growth and Metabolic Changes after Antiretroviral Initiation in South African Children	2021	Pediatr. Infect. Dis. J.	Masi-Leon et al	No INSTI arm

118. Contemporary antiretrovirals and body-mass index: a prospective study of the RESPOND cohort consortium	2021	Lancet HIV	Bansi-Matharu et al	No outcome of interest
119. Tenofovir Alafenamide (TAF) is an Independent Risk Factor for Hyperlipidemia in Persons with Human Immunodeficiency Virus (HIV) on Antiretroviral Therapy (ART)	2021	Open Forum Infect. Dis.	Patel et al	Poster
120. Evaluation of the Incidence of Hypertension, Diabetes, and Hyperlipidemia in Patients on Antiretroviral Therapy	2021	Open Forum Infect. Dis.	Idrees et al	Poster
121. Short- and Long-Term Metabolic Changes in Virologically Suppressed Patients Switching from TDF to TAF Containing Antiretroviral Therapy	2021	Open Forum Infect. Dis.	Schafer et al	Poster
122. Incidence of metabolic complications among treatment-naI&Die	2021	Open Forum Infect. Dis.	Daar et al	All arms contain DTG
123. A Real-world Study Assessing the Risk of Lipid Changes and Other Metabolic Effects Associated with Integrase Inhibitor-based Antiretroviral Therapy	2021	Open Forum Infect. Dis.	Gruss et al	Poster
124. Stratifying the risk of NAFLD in patients with HIV under combination antiretroviral therapy (cART)	2021	eClinicalMedicine	Bischoff et al	No outcome of interest
125. Implications of weight gain with newer anti-retrovirals: 10-year predictions of cardiovascular disease and diabetes	2021	AIDS	McCann et al	No outcome of interest
126. Association of HIV-1 Infection and Antiretroviral Therapy With Type 2 Diabetes in the Hispanic Population of the Rio Grande Valley, Texas, USA	2021	Front. Med.	Lopez et al	Cross sectional study
127. Weight and metabolic changes after switching from tenofovir disoproxil fumarate to tenofovir alafenamide in people living with HIV: A Cohort Study	2021	Ann. Intern. Med.	Surial et al	No INSTI arm
128. Changes in Homeostatic Model Assessment for Insulin Resistance (HOMA-IR) Index in Treated HIV-1 Infected People on Virological Suppression Who Switched to a Different [illegible]	2021	J. Acquired Immune Defic. Syndr.	Muccini et al	Cohort already reported on in another study by
130. Lower Cumulative Antiretroviral Exposure in People Living with HIV and Diabetes Mellitus	2020	J. Acquired Immune Defic. Syndr.	Mann et al	No outcome of interest
131. A prospective, randomized clinical trial of antiretroviral therapies on carotid wall thickness	2015	AIDS	Stein et al	No raw data to calculate relative risks
132. Metabolic changes in the patients on second-line highly active antiretroviral therapy (HAART): A prospective cohort study from north India.	2020	Journal of Family Medicine & Primary Care	Meena et al	No INSTI arm
133. Integrase Strand Transfer Inhibitors are associated with incident diabetes mellitus in poeple with HIV	2022	Clinical infectious Diseases	O'Halloran et al	INSTIS arm contains PI and NNRTI

Table S7: Meta-regression* analysis of study and HIV-related variables on the pooled effect estimate

Variable	Number of studies included	Estimate	LCI	UCI	I^2 Residual heterogeneity (%)	R^2 Amount of heterogeneity accounted for (%)
**Publication year**	13	0.02	-0.08	0.13	51.2	0
**Proportion of black population**	11	0.002	-0.01	0.01	28.4	0
**Male proportion**	12	-0.002	-0.02	0.01	24	0
**Follow-up duration (per year)**	13	-0.11	-0.18	-0.04	0%	100%

*Only when ≥10 studies are included. LCI=lower confidence interval. UCI=upper confidence interval. Only follow-up duration is significantly associated with the pooled effect estimate in 13 studies, every additional year carries an 11% decrease in the pooled risk.

Sub-analysis forest plots for the association of integrase inhibitor use with incident diabetes mellitus with or without metabolic syndrome. In all Forest plots, the black polygon represents the summary measure of the random effects meta-analysis for each subgroup analysis. RR= relative risk, INSTIs= integrase strand transfer inhibitors, PIs=protease inhibitors, NNRTIs=non nucleotide reverse transcriptase inhibitors.

Figure S1: Sub-analysis by non-INSTIs regimen in the control group (protease inhibitors and non-nucleotide reverse transcriptase inhibitors)

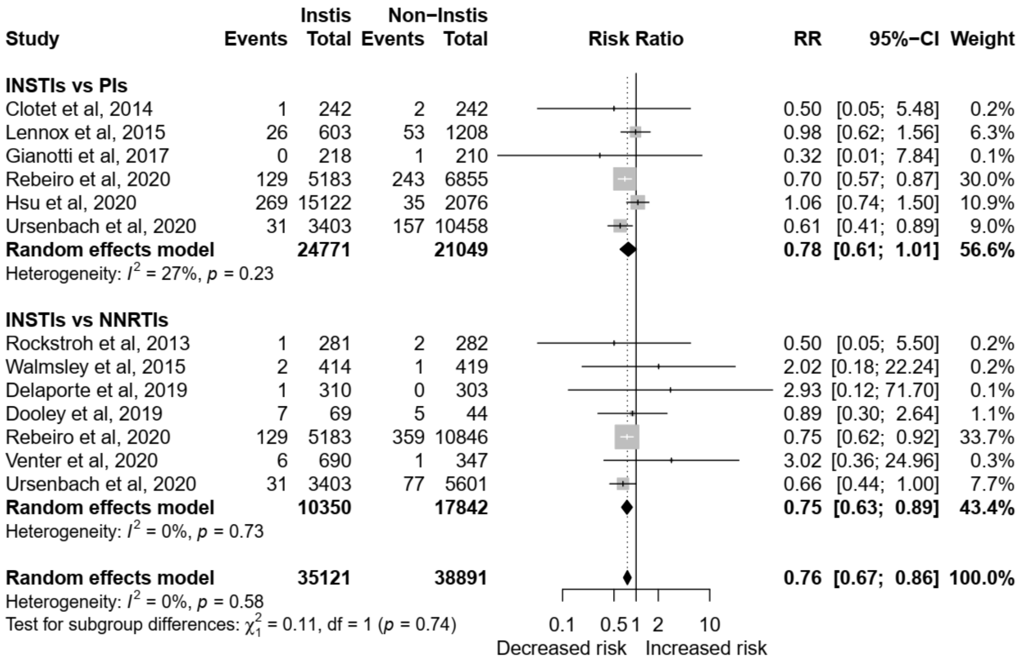

Mulindwa F, *et al. BMJ Open Diab Res Care* 2024; 11:e003136. doi: 10.1136/bmjdrc-2022-003136

Figure S2: Sub-analysis by follow-up duration

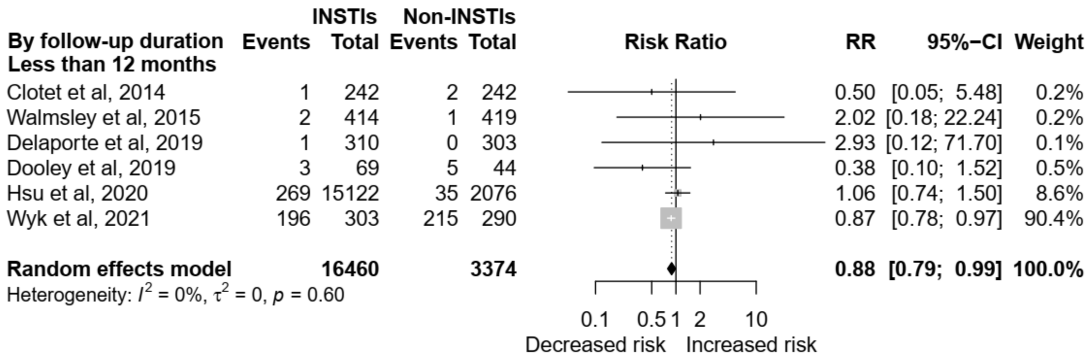

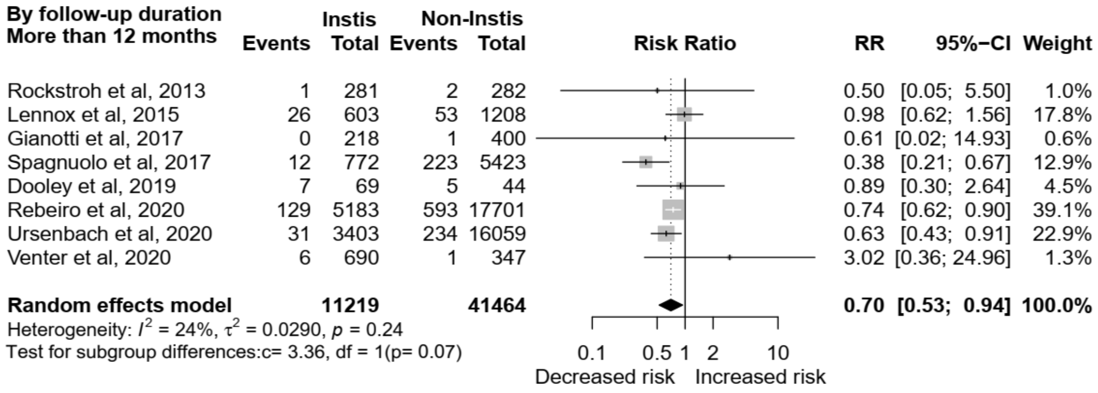

Mulindwa F, et al. BMJ Open Diab Res Care 2024; 11:e003136. doi: 10.1136/bmjdrc-2022-003136

Figure S3: Sub-analysis geographical origin of the study population

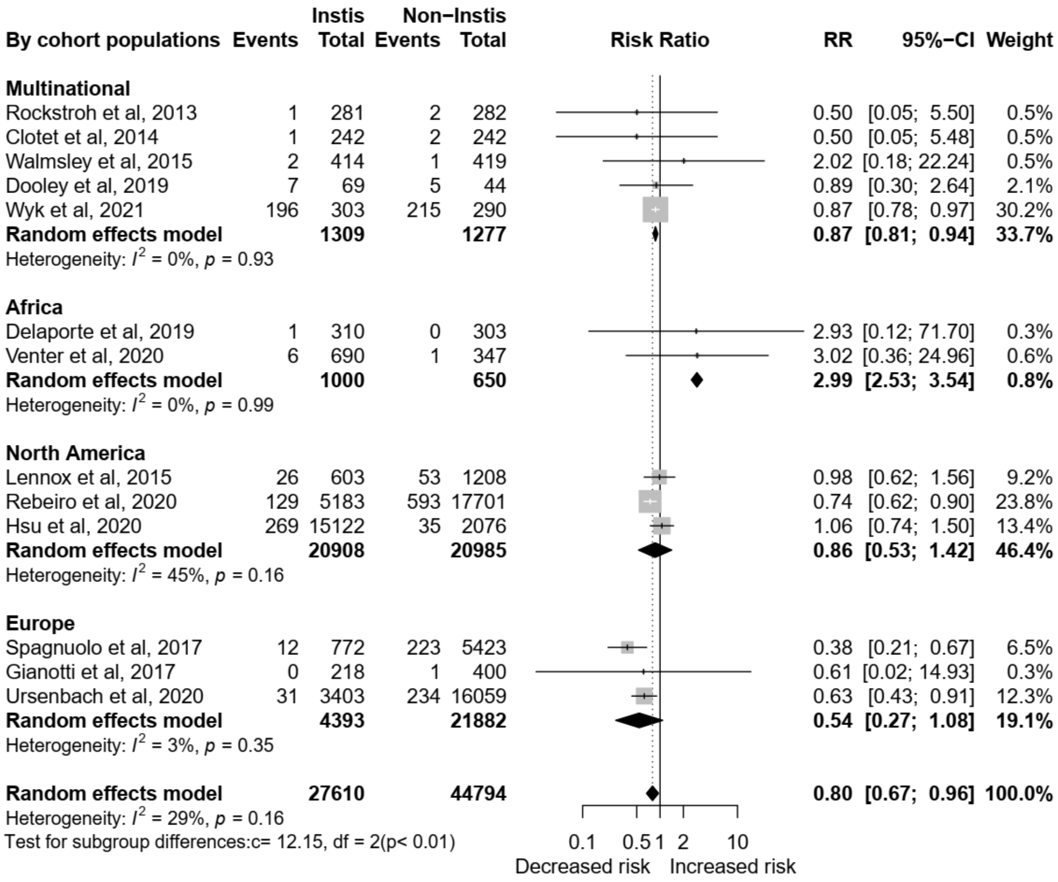

Mulindwa F, *et al. BMJ Open Diab Res Care* 2024; 11:e003136. doi: 10.1136/bmjdrc-2022-003136

Figure S4: Sub-analysis by ART status at baseline

By ART status at baseline	Instis Events	Instis Total	Non−Instis Events	Non−Instis Total	RR	95%−CI	Weight
**ART experienced**							
Hsu et al, 2020	171	8595	25	1109	0.88	[0.58; 1.34]	11.0%
Wyk et al, 2021	18	305	21	280	0.79	[0.43; 1.45]	5.1%
**Random effects model**		**8900**		**1389**	**0.85**	**[0.43; 1.68]**	**16.2%**
Heterogeneity: $I^2 = 0\%$, $p = 0.76$							
**ART naïve**							
Rockstroh et al, 2013	1	281	2	282	0.50	[0.05; 5.50]	0.3%
Clotet et al, 2014	1	242	2	242	0.50	[0.05; 5.48]	0.3%
Lennox et al, 2015	26	603	53	1208	0.98	[0.62; 1.56]	9.0%
Walmsley et al, 2015	2	414	1	419	2.02	[0.18; 22.24]	0.3%
Gianotti et al, 2017	0	218	1	400	0.61	[0.02; 14.93]	0.2%
Delaporte et al, 2019	1	310	0	303	2.93	[0.12; 71.70]	0.2%
Dooley et al, 2019	3	69	5	44	0.38	[0.10; 1.52]	1.0%
Rebeiro et al, 2020	129	5183	593	17701	0.74	[0.62; 0.90]	53.8%
Hsu et al, 2020	98	6527	10	967	1.45	[0.76; 2.77]	4.5%
Ursenbach et al, 2020	31	3403	234	16059	0.63	[0.43; 0.91]	13.7%
Venter et al, 2020	6	690	1	347	3.02	[0.36; 24.96]	0.4%
**Random effects model**		**17940**		**37972**	**0.78**	**[0.65; 0.94]**	**83.8%**
Heterogeneity: $I^2 = 3\%$, $p = 0.42$							
**Random effects model**		**26840**		**39361**	**0.79**	**[0.68; 0.91]**	**100.0%**
Heterogeneity: $I^2 = 0\%$, $p = 0.56$							
Test for subgroup differences:c= 1.13, df = 1(p= 0.29)							

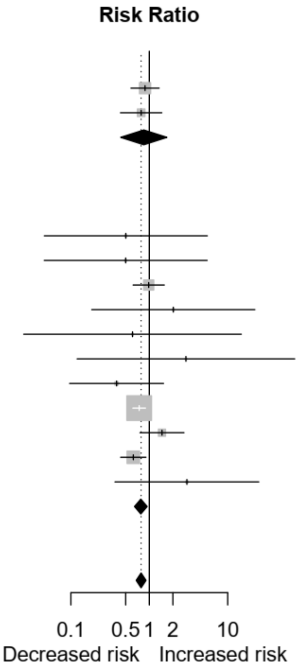

Mulindwa F, *et al. BMJ Open Diab Res Care* 2024; 11:e003136. doi: 10.1136/bmjdrc-2022-003136

Figure S5: Sub-analysis by study design

By study design	Instis Events	Instis Total	Non−Instis Events	Non−Instis Total	RR	95%−CI	Weight
**Randomised controlled trials**							
Rockstroh et al, 2013	1	281	2	282	0.50	[0.05; 5.50]	0.5%
Clotet et al, 2014	1	242	2	242	0.50	[0.05; 5.48]	0.5%
Walmsley et al, 2015	2	414	1	419	2.02	[0.18; 22.24]	0.5%
Lennox et al, 2015	26	603	53	1208	0.98	[0.62; 1.56]	9.2%
Delaporte et al, 2019	1	310	0	303	2.93	[0.12; 71.70]	0.3%
Dooley et al, 2019	7	69	5	44	0.89	[0.30; 2.64]	2.1%
Venter et al, 2020	6	690	1	347	3.02	[0.36; 24.96]	0.6%
Wyk et al, 2021	196	303	215	290	0.87	[0.78; 0.97]	30.2%
**Random effects model**		**2912**		**3135**	**0.88**	**[0.81; 0.96]**	**43.8%**
Heterogeneity: I^2 = 0%, p = 0.89							
**Cohort**							
Spagnuolo et al, 2017	12	772	223	5423	0.38	[0.21; 0.67]	6.5%
Gianotti et al, 2017	0	218	1	400	0.61	[0.02; 14.93]	0.3%
Rebeiro et al, 2020	129	5183	593	17701	0.74	[0.62; 0.90]	23.8%
Hsu et al, 2020	269	15122	35	2076	1.06	[0.74; 1.50]	13.4%
Ursenbach et al, 2020	31	3403	234	16059	0.63	[0.43; 0.91]	12.3%
**Random effects model**		**24698**		**41659**	**0.69**	**[0.44; 1.10]**	**56.2%**
Heterogeneity: I^2 = 60%, p = 0.04							
**Random effects model**		**27610**		**44794**	**0.80**	**[0.67; 0.96]**	**100.0%**
Heterogeneity: I^2 = 29%, p = 0.16							
Test for subgroup differences: χ^2_1 = 1.99, df = 1 (p = 0.16)							

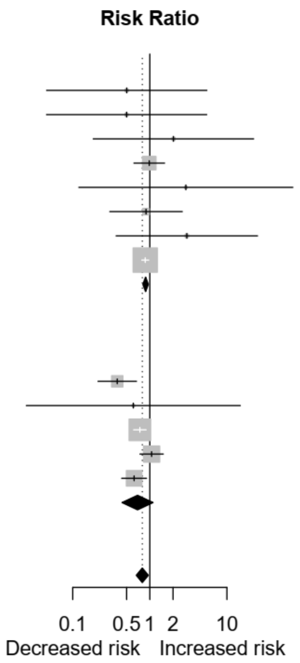

Mulindwa F, *et al. BMJ Open Diab Res Care* 2024; 11:e003136. doi: 10.1136/bmjdrc-2022-003136

Figure S6. Assessment of publication bias by funnel plot asymmetry test for 13 studies meta-analysed for incident insulin resistance and/or diabetes in INSTIs compared to non-INSTIs.

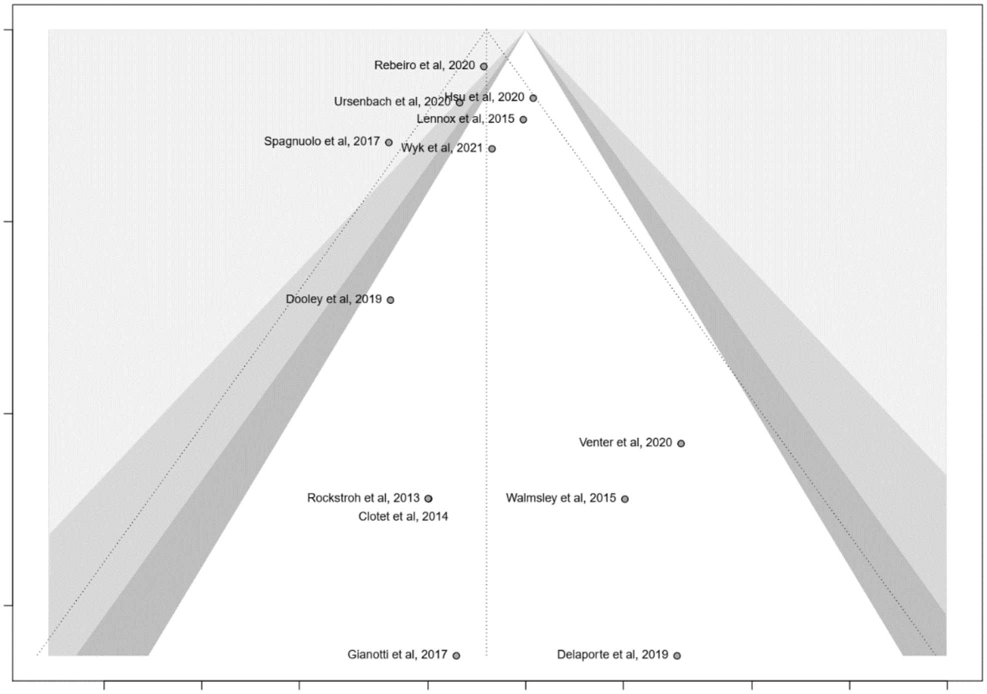

Egger's test for the intercept:

Intercept = 0.093 (95% CI -0.84 – 1.03, P=0.85)
There is no funnel plot asymmetry by Egger's test.

Figure S7. Mean changes in HOMA-IR from baseline in INSTIs group compared to overall non-INSTIs, NNRTIs and PIs groups.

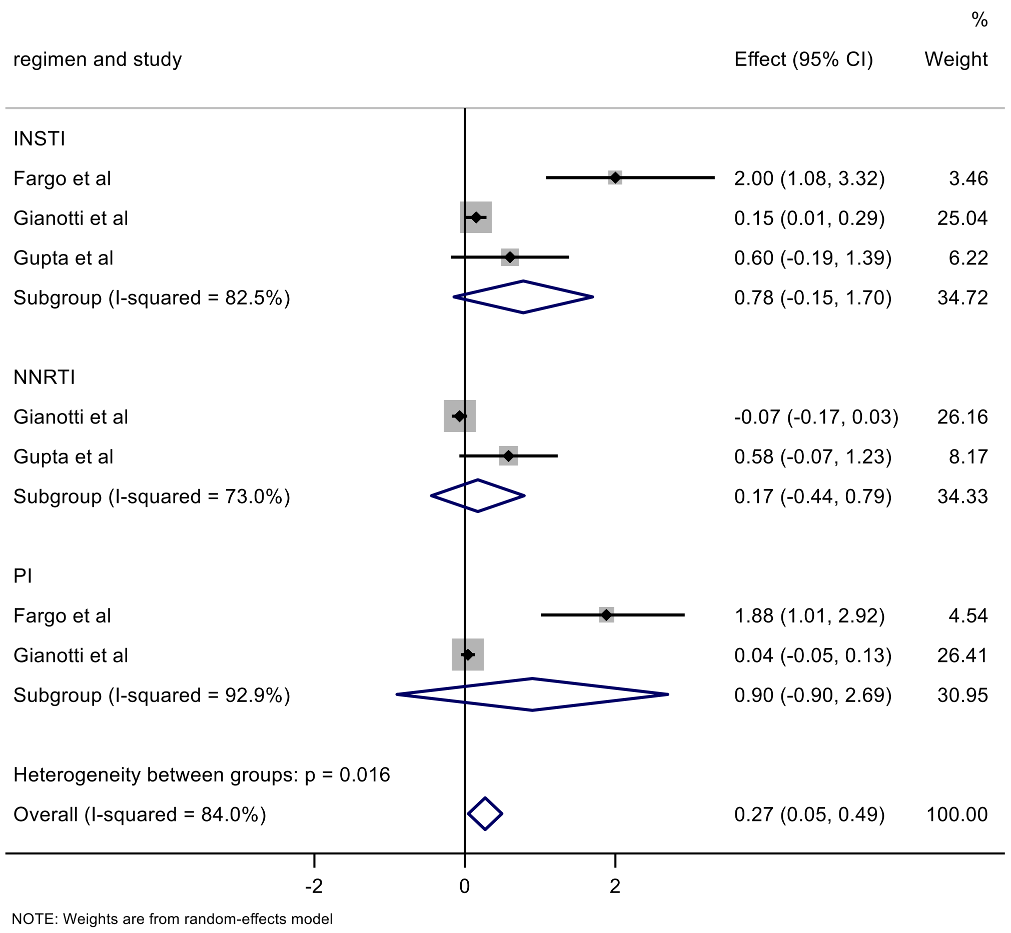

Abbreviations: HOMA IR- Homeostatic model for insulin resistance index. INSTIs=integrase strand transfer inhibitors. NNRTI=non-nucleoside reverse transcriptase inhibitors, PI=protease inhibitors.

Figure S8: Bubble plots for univariable meta-regression on (A) Baseline viral load (B) Age (C) Baseline CD4 cell count

Mulindwa F, *et al. BMJ Open Diab Res Care* 2024; 11:e003136. doi: 10.1136/bmjdrc-2022-003136

A)

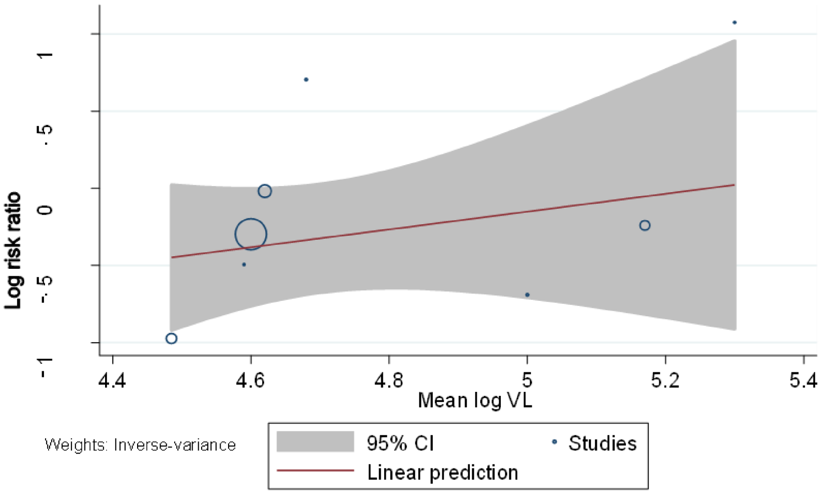

P= 0·434

VL- viral load

B)

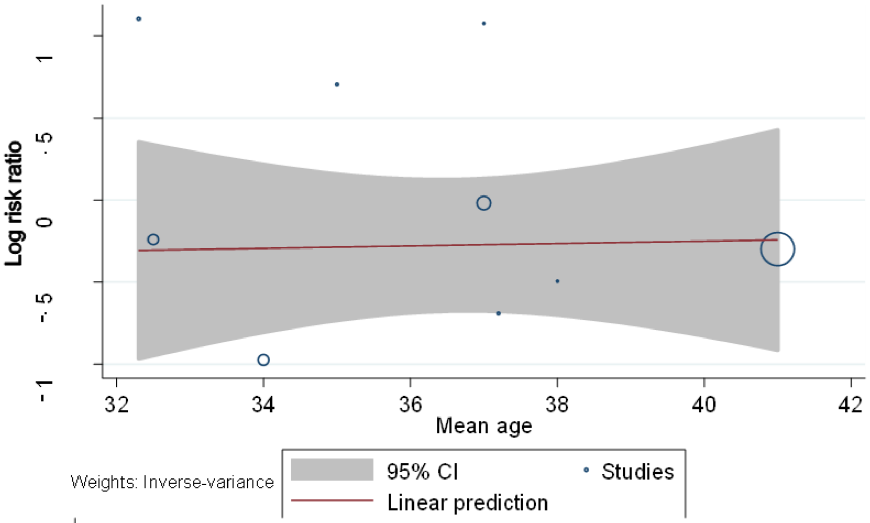

P=

Age
0·907

C)

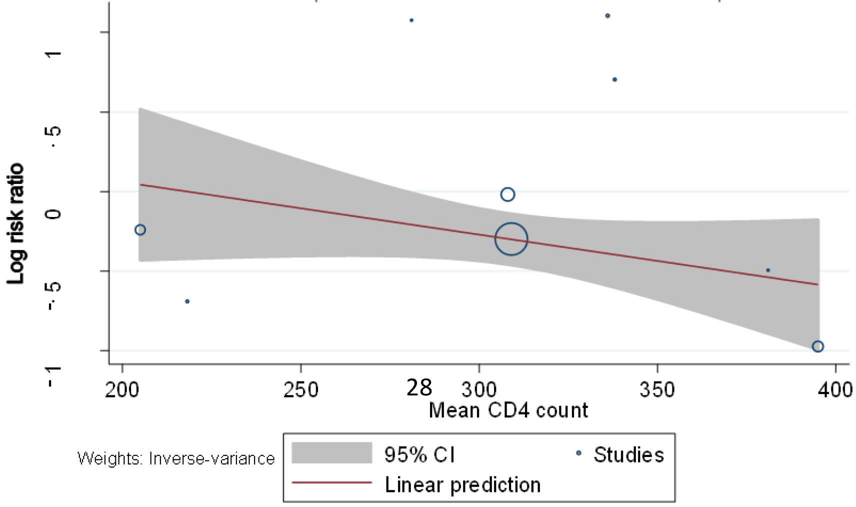

Baseline CD4 count **P= 0·133**

Visual trends suggest a heightened risk of diabetes with exposure to INSTIs in patients with a high baseline viral load (A) and low $CD4^+$ cell count (C). However, this heightened risk was not statistically significant. There was no trend suggestive of age affecting the association between integrase strand transfer inhibitors and incident diabetes.

Figure S9: Bubble plot for univariable meta-regression on follow-up time

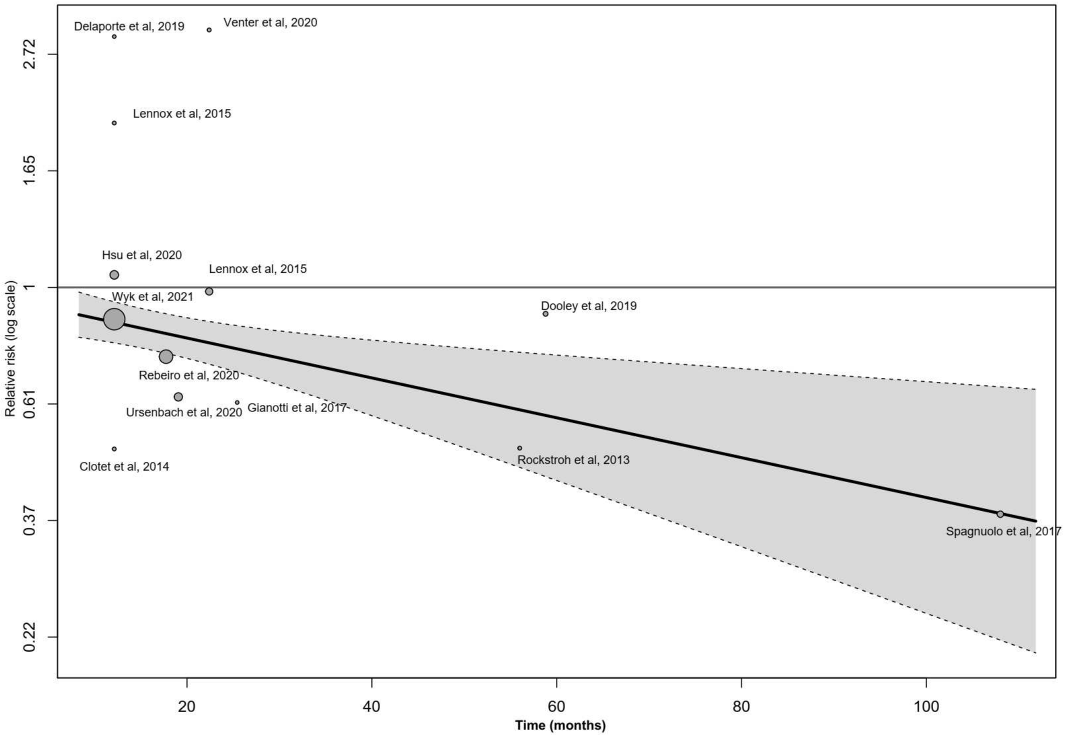

As demonstrated, the risk of developing diabetes with exposure to INSTIS decreased significantly with longer duration of follow-up.

See Figure S2 for the pooled risk by follow-up time (12 months).

Figure S10: Influence analysis*by leave one-out-method for 13 studies pooled in the meta-analysis sorted by effect size.

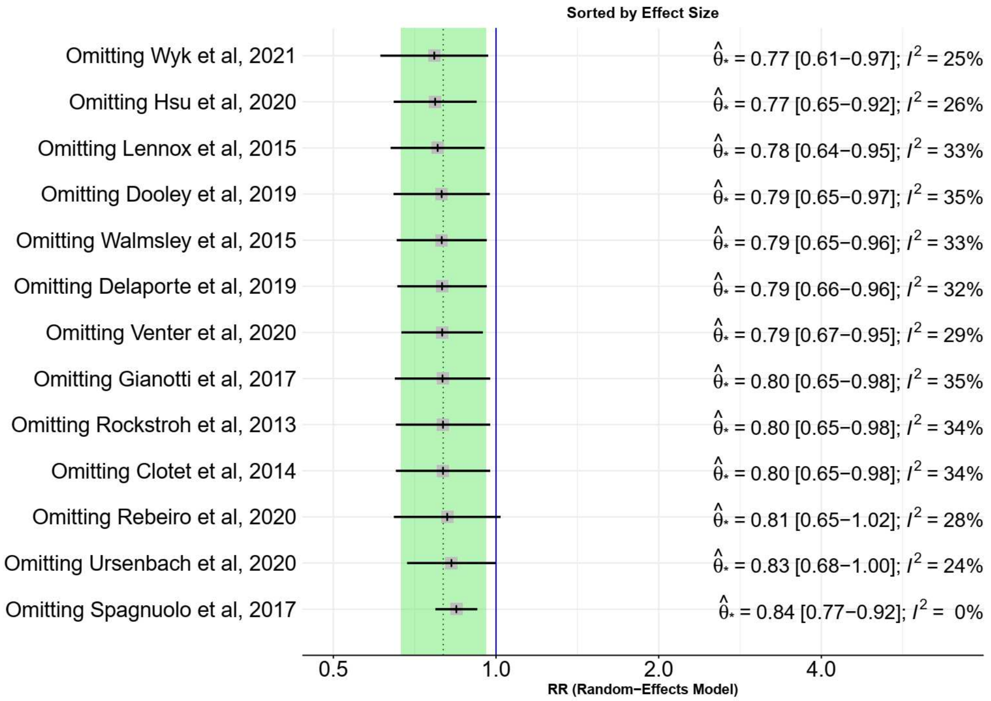

Abbreviations: θ=effect size; I2=heterogeneity; RR=relative risk. In Influence analysis the pooled effect estimate is calculated while omitting one study at a time to detect the individual impact of each study. For e.g., omitting Spagnuolo et al.2017 will yield a pooled relative risk of the remaining 12 studies= 0.84 with 95% CI (0.77-0.92) with minimal heterogeneity I2=0%.

Mulindwa F, *et al. BMJ Open Diab Res Care* 2024; 11:e003136. doi: 10.1136/bmjdrc-2022-003136

Figure S11: Baujat plot*showing each study contribution to the overall heterogeneity of the meta-analysis (studies n=13)

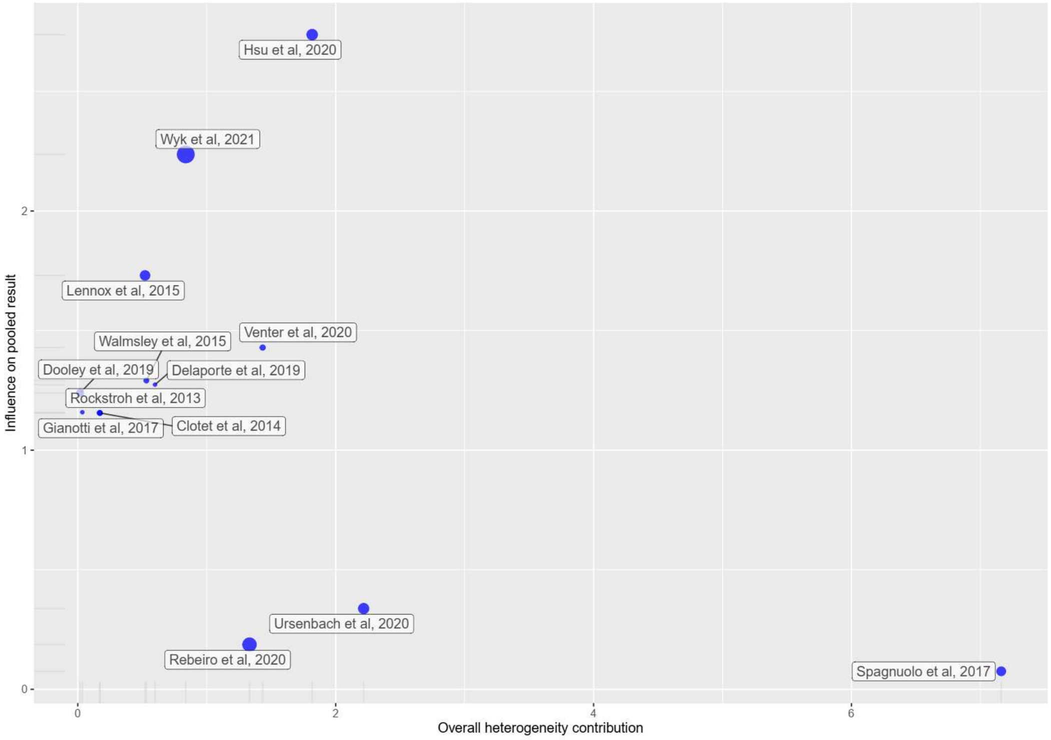

The contribution of each study to the overall heterogeneity is plotted on the X axis, and each study influence on the pooled effect estimate is plotted on the Y axis. Study by Spagnuolo et al. added the most to heterogeneity but with minimal impact on the effect size due to its small sample size.

**Baujat B, Mahe C, Pignon JP, Hill C. A graphical method for exploring heterogeneity in meta-analyses: application to a meta-analysis of 65 trials. Stat Med.2002 Sep 30;21(18):2641-52. doi:10.1002/sim.1221*

Continuation of manuscript references:

61 McLaughlin M, Walsh S, Galvin S. Dolutegravir-induced hyperglycaemia in a patient living with HIV. *J Antimicrob Chemother* 2018;**73**:258–60. doi:https://dx.doi.org/10.1093/jac/dkx365

62 Eckard AR, McComsey GA. Weight gain and integrase inhibitors. *Curr Opin Infect Dis* 2020;**33**:10–9. doi:10.1097/QCO.0000000000000616

63 Sax PE, Erlandson KM, Lake JE, *et al.* Weight Gain Following Initiation of Antiretroviral Therapy: Risk Factors in Randomized Comparative Clinical Trials. *Clinical Infectious Diseases* 2020;**71**:1379–89. doi:10.1093/CID/CIZ999

64 Todowede OO, Mianda SZ, Sartorius B. Prevalence of metabolic syndrome among HIV-positive and HIV-negative populations in sub-Saharan Africa - A systematic review and meta-analysis 11 Medical and Health Sciences 1117 Public Health and Health Services. *Syst Rev* 2019;**8**:1–17. doi:10.1186/S13643-018-0927-Y/TABLES/6

65 Ghislain M, Bastard JP, Meyer L, *et al.* Late Antiretroviral Therapy (ART) Initiation Is Associated with Long-Term Persistence of Systemic Inflammation and Metabolic Abnormalities. *PLoS One* 2015;**10**:144317. doi:10.1371/JOURNAL.PONE.0144317

66 Sax PE, Erlandson KM, Lake JE, *et al.* Weight Gain Following Initiation of Antiretroviral Therapy: Risk Factors in Randomized Comparative Clinical Trials. *Clin Infect Dis* 2020;**71**:1379. doi:10.1093/CID/CIZ999

67 Birabaharan M, Strunk A, Kaelber DC, *et al.* Sex differences in type 2 diabetes mellitus prevalence among persons with HIV. *AIDS* 2022;**36**:383–9. doi:10.1097/QAD.0000000000003127

68 Prioreschi A, Munthali RJ, Soepnel L, *et al.* Incidence and prevalence of type 2 diabetes mellitus with HIV infection in Africa: a systematic review and meta-analysis. *BMJ Open* 2017;**7**:e013953. doi:10.1136/BMJOPEN-2016-013953

69 Spinelli MA, Hessol NA, Schwarcz SK, *et al.* Disparities in Integrase Inhibitor Usage in the Modern HIV Treatment Era: A Population-Based Study in a US City. *Open Forum Infect Dis* 2021;**8**. doi:10.1093/OFID/OFAB139

70 Naito T, Mori H, Fujibayashi K, *et al.* Analysis of antiretroviral therapy switch rate and switching pattern for people living with HIV from a national database in Japan. *Scientific Reports 2022 12:1* 2022;**12**:1–11. doi:10.1038/s41598-022-05816-5

71 Version. Division of AIDS (DAIDS) Table for Grading the Severity of Adult and Pediatric Adverse Events. 2014.
